# Regional analysis of inflammation and contractile function in reperfused acute myocardial infarction by in vivo ^19^F cardiovascular magnetic resonance in pigs

**DOI:** 10.1007/s00395-022-00928-5

**Published:** 2022-04-07

**Authors:** Florian Bönner, M. Gastl, F. Nienhaus, M. Rothe, A. Jahn, S. Pfeiler, U. Gross, H.-P. Schultheiss, B. Ibanez, S. Kozerke, J. Szendroedi, M. Roden, R. Westenfeld, J. Schrader, U. Flögel, G. Heusch, M. Kelm

**Affiliations:** 1 Division of Cardiology, Pulmonology, and Vascular Medicine, Medical Faculty Heinrich Heine University, Moorenstr. 5, 40225 Düsseldorf, Germany; 2 Institute for Clinical Diabetology, German Diabetes Center, Leibniz Center for Diabetes Research, Düsseldorf, Germany; 3 German Center for Diabetes Research, Partner, Düsseldorf, Germany; 4 Central Animal Research Facility, Heinrich Heine University, Düsseldorf, Germany; 5 Institute for Cardiac Diagnostics and Therapy (IKDT), Berlin, Germany; 6 Centro Nacional de Investigaciones Cardiovasculares Carlos III (CNIC), Madrid, Spain; 7 IIS-Fundación Jiménez Díaz Hospital, Madrid, Spain; 8 CIBERCV, Madrid, Spain; 9 Institute for Biomedical Engineering, University and ETH Zurich, Zurich, Switzerland; 10 Division of Endocrinology and Diabetology, Medical Faculty, Heinrich Heine University, Düsseldorf, Germany; 11 Department of Molecular Cardiology, Heinrich Heine University, Düsseldorf, Germany; 12 Experimental Cardiovascular Imaging, Department of Molecular Cardiology, Heinrich Heine University, Düsseldorf, Germany; 13 Institute for Pathophysiology, West German Heart and Vascular Center, University of Essen Medical School, Essen, Germany; 14 Cardiovascular Research Institute Düsseldorf (CARID), Medical Faculty, Heinrich Heine University, Düsseldorf, Germany

**Keywords:** Cardiovascular magnetic resonance, Inflammation, Large animal models, Monocytes/macrophages, Myocardial infarction

## Abstract

Inflammatory cell infiltration is central to healing after acute myocardial infarction (AMI). The relation of regional inflammation to edema, infarct size (IS), microvascular obstruction (MVO), intramyocardial hemorrhage (IMH), and regional and global LV function is not clear. Here we noninvasively characterized regional inflammation and contractile function in reperfused AMI in pigs using fluorine (^19^F) cardiovascular magnetic resonance (CMR). Adult anesthetized pigs underwent left anterior descending coronary artery instrumentation with either 90 min occlusion (*n* = 17) or without occlusion (sham, *n* = 5). After 3 days, in surviving animals a perfluorooctyl bromide nanoemulsion was infused intravenously to label monocytes/macrophages. At day 6, in vivo ^1^H-CMR was performed with cine, T2 and T2* weighted imaging, T2 and T1 mapping, perfusion and late gadolinium enhancement followed by ^19^F-CMR. Pigs were sacrificed for subsequent ex vivo scans and histology. Edema extent was 35 ± 8% and IS was 22 ± 6% of LV mass. Six of ten surviving AMI animals displayed both MVO and IMH (3.3 ± 1.6% and 1.9 ± 0.8% of LV mass). The ^19^F signal, reflecting the presence and density of monocytes/macrophages, was consistently smaller than edema volume or IS and not apparent in remote areas. The ^19^F signal-to-noise ratio (SNR) > 8 in the infarct border zone was associated with impaired remote systolic wall thickening. A whole heart value of ^19^F integral (^19^F SNR × milliliter) > 200 was related to initial LV remodeling independently of edema, IS, MVO, and IMH. Thus, ^19^F-CMR quantitatively characterizes regional inflammation after AMI and its relation to edema, IS, MVO, IMH and regional and global LV function and remodeling.

**Supplementary Information:**

The online version contains supplementary material available at 10.1007/s00395-022-00928-5.

## Introduction

Acute myocardial infarction (AMI) and ischemic heart disease remain the leading causes of death and heart failure [43]. All-cause mortality and rehospitalization for heart failure within 1 year after AMI are critically dependent on infarct size (IS) [36]. Microvascular obstruction (MVO) and intramyocardial hemorrhage (IMH), two major features of myocardial reperfusion injury [15, 16], are of additional value in the prediction of late adverse regional and global left ventricular (LV) remodeling and mortality [6, 25, 29]. The processes of evolving edema, infarction, MVO, and IMH in reperfused AMI are dynamic and interact with inflammatory infiltration and the initiation of healing and repair [9, 19]; the repair relies on immune cell recruitment, most importantly monocytes and derived macrophages [37]. Monocytes/macrophages are attracted to the infarcted myocardium in a time- and region-dependent manner, as evidenced by histology in human myocardium [42]. Whereas inflammation is necessary for healing and repair, excessive inflammation is detrimental [14], and therapeutic modulation of inflammation after AMI might reduce patient morbidity and mortality [12, 38]. Multiparametric ^1^H-based cardiovascular magnetic resonance (^1^H-CMR) has become the gold standard for the noninvasive assessment of IS, MVO, IMH, and LV function after AMI [19]. However, there is an unmet need to visualize and quantify the spatial and temporal cellular inflammatory patterns in vivo.

Using experimental high-field (9.4 T) CMR, we established fluorine (^19^F)-CMR to monitor monocytes/macrophages in mouse models [10]. ^19^F MRI noninvasively monitors specifically infiltrating monocytes due to their rapid and specific uptake of intravenously applied perfluorocarbon nanoemulsions [3, 26]. Recently, we have demonstrated the technical feasibility of this ^19^F-CMR approach also in clinical scanners (i.e. field strength of 3 T) [4, 33]. In the present study, we adapted this approach to a pig model of reperfused AMI which permits a spatially resolved regional analysis of inflammatory infiltration and its relation to edema, IS, MVO, IMH as well its impact on regional and global LV contractile function.

## Material and methods

### Reperfused acute myocardial infarction in pigs

**Fig. 1:**
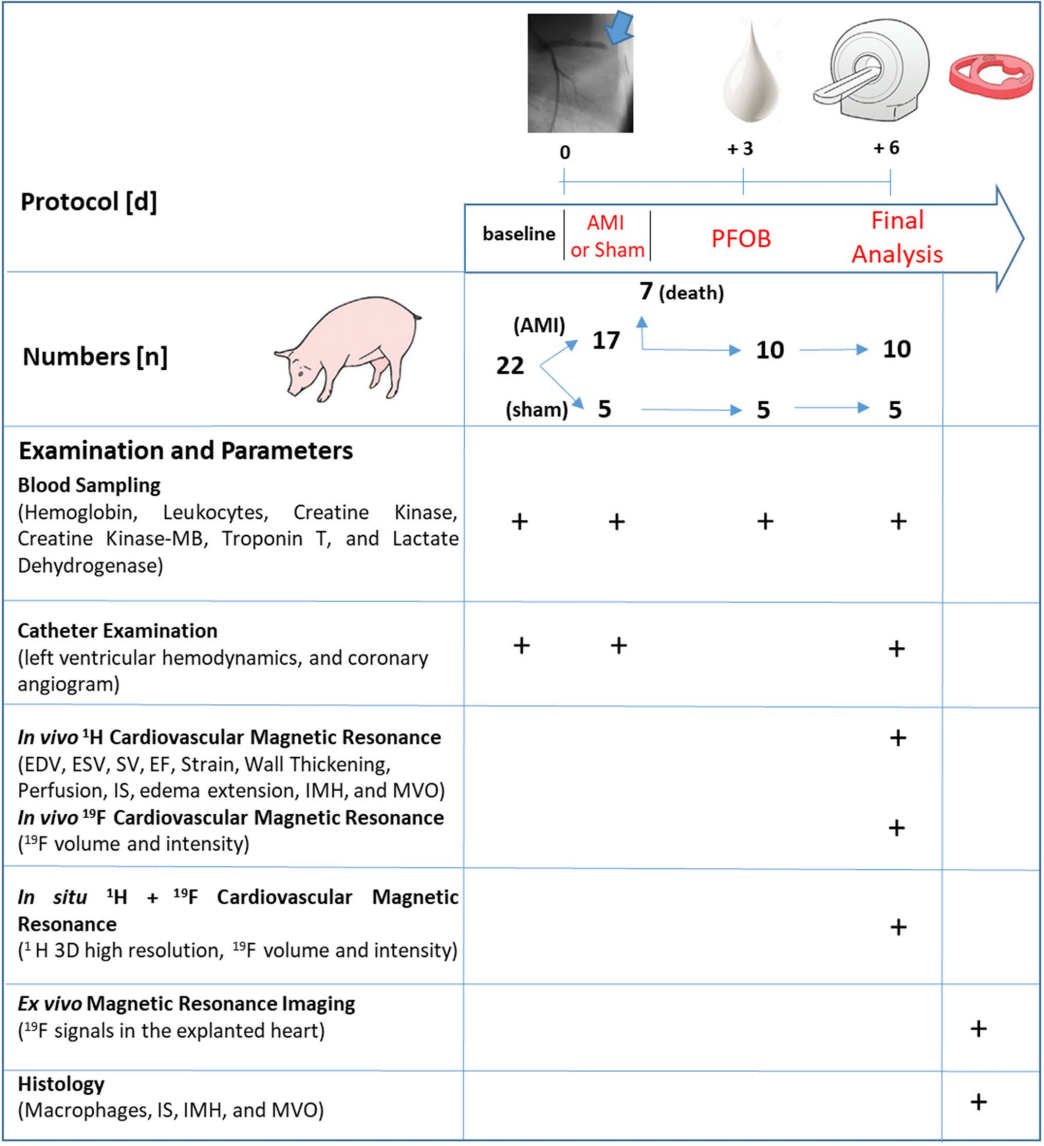
Study design and protocol. Seventeen pigs were subjected to acute myocardial infarction (AMI), and 5 pigs underwent sham surgery. A perfluorooctyl bromide (PFOB) nanoemulsion was administered on day 3 after AMI. Fifteen pigs underwent in vivo cardiovascular magnetic resonance (CMR + ^19^F) with analysis of volumes, contractile function and tissue characterization. In situ and ex vivo magnetic resonance imaging, followed by histology, was performed for validation purposes. *EDV* end diastolic volume, *ESV* end systolic volume, *SV* stroke volume, *EF* ejection fraction, *IS* infarct size, *IMH* intramyocardial hemorrhage, *MVO* microvascular obstruction

Experiments were performed in 22 adult female Aachen minipigs [27] with a mean body weight of 67 ± 9 kg, in accordance with the national guidelines on animal care and approval by the ‘Landesamt für Natur, Umwelt- und Verbraucherschutz’ (L84-02.04.2013.A437 and L84-02.04.2016.A322). The experimental protocol is displayed in Fig. 1. Myocardial infarction was induced in pentobarbital-isoflurane-anesthetized closed-chest pigs by 90 min of pressure-wire-controlled left anterior descending (LAD) coronary artery occlusion [33], for details see supplemental material & methods and supplemental Figure I. In 5 sham animals the LAD was instrumented but not subjected to balloon occlusion.

### Production, quality control and application of perfluorooctyl bromide nanoemulsion

The perfluorooctyl bromide nanoemulsion (PFOB) was produced according to established protocols [4, 33]. A detailed description of the production, quality control, stability assessment, blood kinetics and phagocytosis of PFOB is provided in the supplemental Figures II and III. PFOB was infused intravenously on day 3 after AMI with 5 ml/kg body weight and a rate of 80 ml/h (Fig. 1) in all surviving animals (*n* = 10 AMI and *n* = 5 sham) according to previous protocols [4].

### ^1^H and ^19^F cardiovascular magnetic resonance data acquisition and histology

After proof of LAD patency 6 days after AMI by invasive angiography, CMR was performed using a whole-body 3.0-T Achieva X-series MR scanner (Philips Healthcare, Best, the Netherlands). In vivo CMR was performed according to previously established workflows and protocols for local signal precision with optimized ^19^F sequences [33]. The ^1^H and ^19^F-sequence protocols and details are reported in supplemental Table I. After in vivo ^1^H- and ^19^F-CMR acquisitions, the pigs were euthanized inside the scanner for in situ imaging with arrested hearts. Autopsy was performed, and organs (heart, liver, spleen and sternum) were explanted and stored in 4% paraformaldehyde (PFA) for analysis of histopathology and distribution of PFOB with high resolution ^19^F-CMR. A detailed description of the histopathological protocols and analysis, including the immunohistochemistry of TNFα, is provided in the supplemental material & methods and supplemental figure IV and V*.*

### Cardiovascular magnetic resonance data analysis

Dedicated software (Circle CVI 42, Circle Cardiovascular Imaging Inc., Calgary, AB, Canada) was used for full automatic delineation of ventricular contours, automatic calculation of volumes and analysis of parametric T1 and T2 maps. Due to misalignment of the automated contouring algorithm, significant manual correction of endocardial contours was necessary in 3/10 pigs after AMI. As described in detail in supplemental material & methods and shown in supplemental figure VI, volumes were indexed to body weight to measure end diastolic volume index (EDVi), end systolic volume index (ESVi), stroke volume index (SVi), cardiac index (CI) as well as ejection fraction (EF), global longitudinal strain (GLS), and early longitudinal diastolic strain rate (SRe) [34]. The sphericity volume index (ShVi) was calculated with EDV/ ((*π*/6) * *L*)^3^ [1]. Initial remodeling was defined as > 20% EDVi increase compared to the EDVi of sham-instrumented animals, adapted from studies investigating long-term remodeling after AMI [41]. T2 weighted images and T2 maps were analyzed for the extent of myocardial edema and expressed as % of LV mass or in absolute mass (g). T2* weighted images were analyzed for IMH and expressed as % of LV mass or in milliliters (ml). Contrast-enhanced images were analyzed at first pass for myocardial perfusion and late gadolinium enhancement (LGE) after 10 min for IS (> 5 SD threshold), border zone (BZ) (> 2 < 5 SD threshold) [20] and after 15 min for MVO and expressed as % of LV mass or in milliliters (ml) [34]. Volumes containing ^19^F signals were quantified using the 3D visualization software Amira 4.0 (ThermoFisher Scientific,Waltham,USA), and image fusion of ^1^H/^19^F-datasets was performed with HOROS (Nimble Co LLC,USA). The fused images of all 10 animals which were included in the final analysis are shown in the supplemental figure VII. For quantification of ^19^F-signals, the primary signal in a respective region was corrected according to the coil sensitivity profile as outlined in supplemental figure VIII*.* The signal-to-noise-ratio (SNR) of respective regions was calculated from the ratio of the signal intensity mean of a region-of-interest (ROI) and the standard deviation of a background-noise ROI in the same slice located out of the thorax at comparable coil distance. ^19^F volumes were calculated by applying background subtraction with SNR 7, which was sufficient to subtract all unspecific technical background signals (outside the body). The ^19^F-integral was the product of SNR and volume.

### Statistical analysis

Statistical analysis was performed using SPSS software (version 24.0, IBM, Armonk, NY, US). Unless otherwise stated, continuous variables are presented as the mean ± standard deviation (SD). Differences between the two groups (sham and AMI) were analyzed by Student’s 2-sided unpaired *t* test for normally distributed data and Mann–Whitney *U* test for non-normally distributed data. Pearson’s or Spearman’s correlation were used to assess the relationships between different CMR parameters. Separate linear regression models in pairwise combination with edema, IS, MVO, and IMH were created to adjust the univariate correlations of ^19^F with LV function and volumes for these parameters to test whether ^19^F would independently predict LV function and geometry. ROC analysis was used to identify optimal cut-off values for prediction of initial remodeling (> 20% EDVi increase compared to the sham animals mean) (AUC 0.87) or impaired remote systolic wall thickening (< cohort median of diastolic wall thickening) (AUC 0.78). The respective *p* values are reported in the figures.

## Results

### Acute myocardial infarction and safety of perfluorooctyl bromide nanoemulsion

Twenty-two pigs were included in the study. Baseline and procedural characteristics of sham pigs (*n* = 5), AMI survivors (n = 10) and AMI non-survivors (*n* = 7) are reported in supplemental Tables II and III. There were no differences in baseline characteristics between survivors and non-survivors. The cause of death (*n* = 7) was ventricular fibrillation refractory to defibrillation in all pigs. No pig died beyond 90 min of ischemia. There was no serious adverse side reaction when pigs received PFOB on day 3 after AMI (supplemental Table IV). The blood kinetics of PFOB are given in supplemental Figure III and revealed an almost complete clearance from the circulation 3 days after injection. Ten pigs had a patent LAD on day 6 after AMI on coronary angiography and were included in the CMR study.

### LV function and initial remodeling early after AMI

**Fig. 2:**
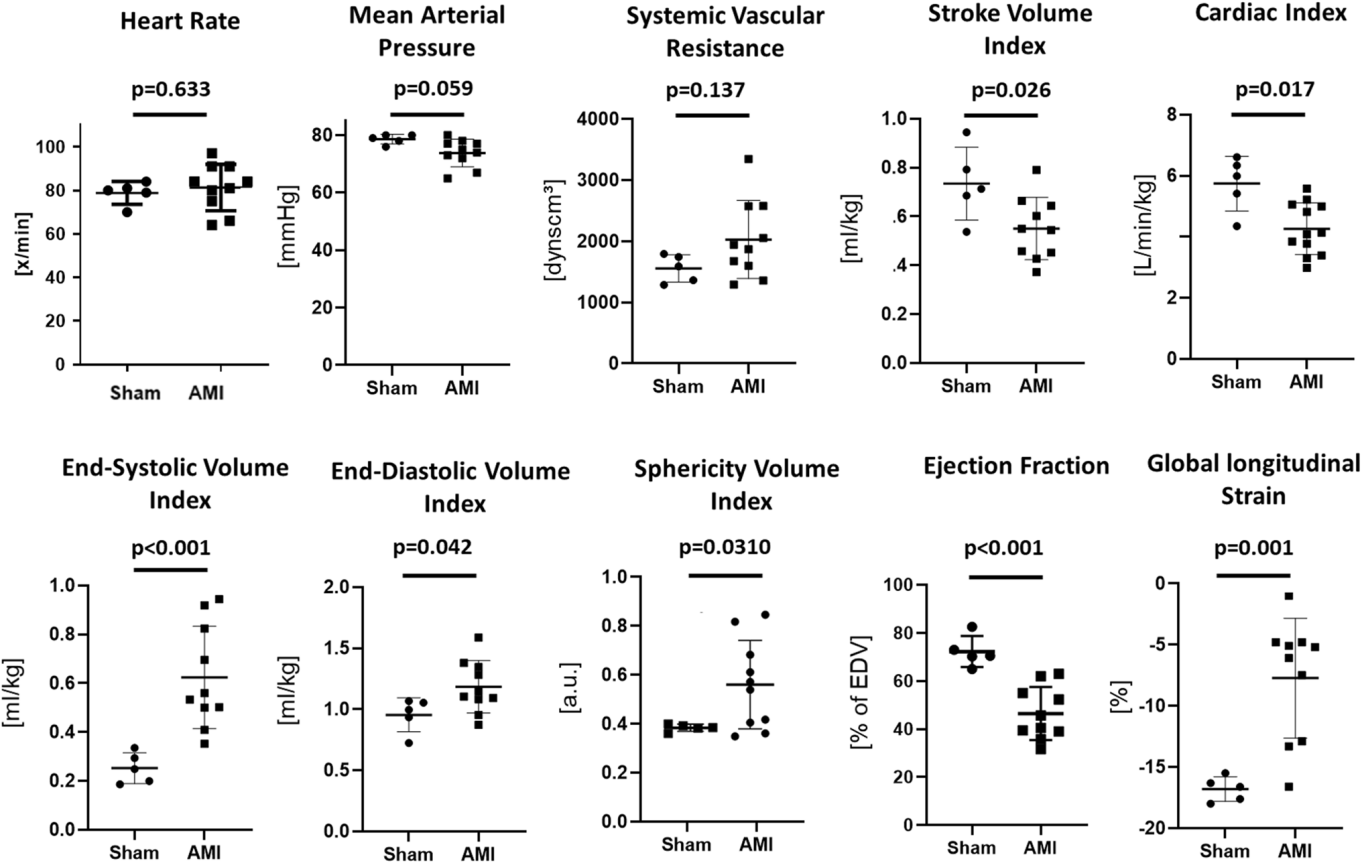
Systemic hemodynamics, left ventricular contractile function and volumes. Quantitative analysis of hemodynamic parameters 6 days after acute myocardial infarction (AMI) compared to the sham procedure. Single values and the mean ± SD as well as the respective *p* values calculated by unpaired Student’s *t* test are given

Edema extent was 35 ± 8% of LV mass and IS was 22 ± 6% of LV mass. Six of 10 surviving pigs had MVO (3.3 ± 1.6% of LV mass), and the same animals also had IMH (1.9 ± 0.8% of LV mass). Figure 2 shows systemic hemodynamic, volumetric and functional data 6 days after AMI. Compared to sham pigs, pigs with AMI had a slight increase in heart rate, a trend toward lower arterial pressure and higher systemic vascular resistance. SVi, CO, EF and LGE were reduced; ESVi, EDVi and ShVi were increased. Thus, the present model provided robust infarction with early LV dysfunction and initial remodeling of varying inter-individual severity.

### ^19^F monocyte/macrophage signals co-register with the infarct area

**Fig. 3:**
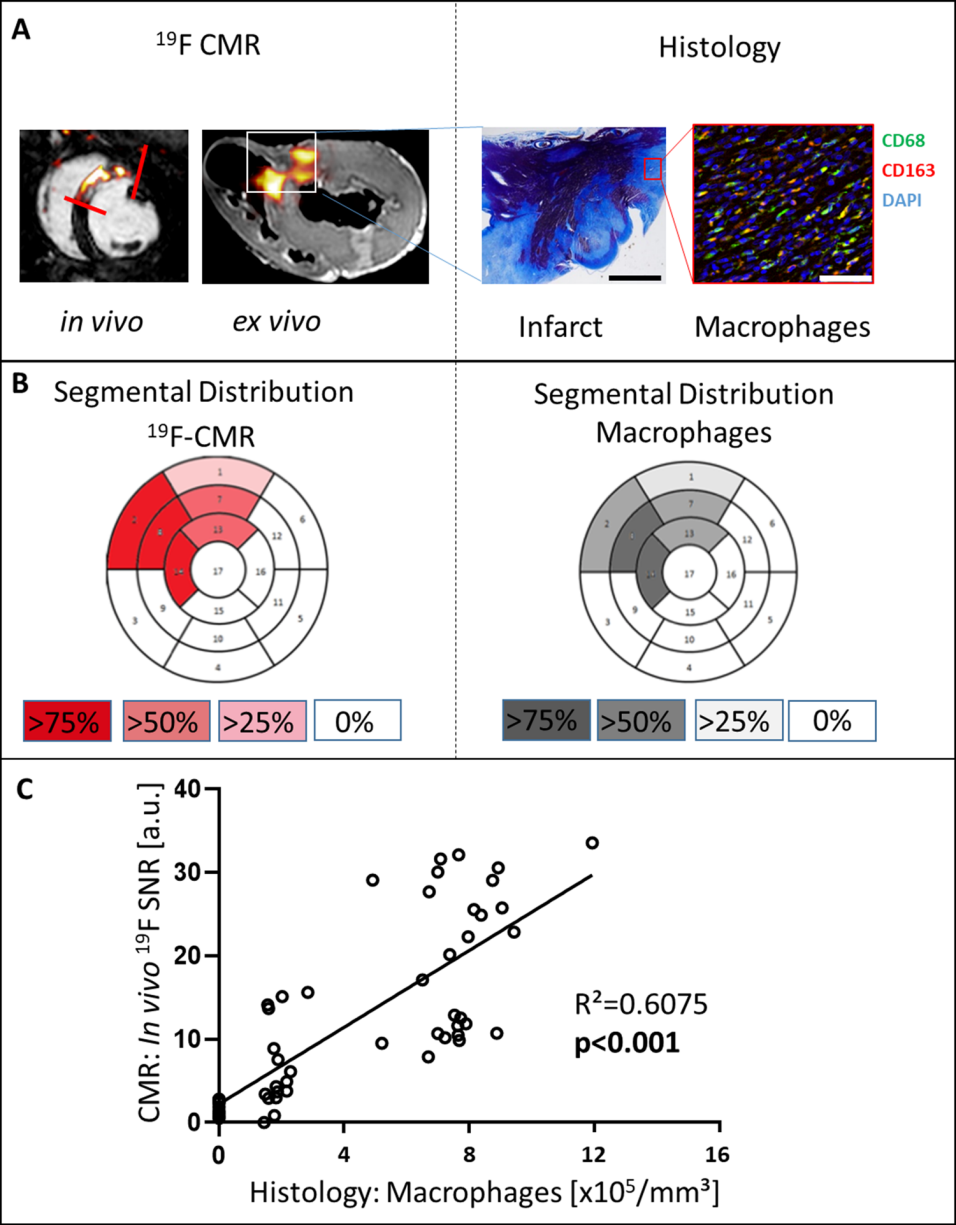
Correlation of in vivo ^19^F signals to macrophage density in histology. **A** Left: ^19^F-signals are heterogeneously distributed in the area of infarction as observed in vivo (red lines) and ex vivo (white box). Right: infarct histology with Masson’s trichrome strain: infarcted tissue = purple, granulation tissue = blue; fluorescent staining with anti-macrophage antibodies: CD68 (green, pseudocolored) and CD163 (red, pseudocolored) at × 40 magnification. Nuclear staining with DAPI (blue). Scale bar 7 mm at Masson`s trichrome strain and 50 µm at fluorescence staining. **B** Segmental analysis with red (^19^F) and black (macrophages) color intensity codes for the percentage of all individuals positive for the respective segment. **C** In vivo ^19^F SNR plotted against histological macrophage density expressed as cells/mm^3^. Shown are data points of all animals. At least 5 regions of interest distributed in at least 2 different slices were considered per animal. If animals had ^19^F signal in more than 5 regions in at least 2 different slices due to their large ^19^F volume, those data points were additionally taken into consideration

The average acquisition time of the 3D-whole heart ^19^F-information was 18 ± 2 min. Figure 3 displays the correlation of local ^19^F-signal intensity and monocyte/macrophage density. ^19^F-signals were restricted to infarcted tissue and its border zone, as delineated by infarct size imaging with LGE, while remote myocardial areas displayed no ^19^F-signal (Fig. 3A, left). The colocalization of the ^19^F-label with monocytes/macrophages was confirmed by histology (Fig. 3A, right and B). The infarct area displayed patchy infiltration with monocytes/macrophages, mirrored by the respective local ^19^F signals. An equal number of M1 (CD68^high^ CD163 ^low^) and M2 (CD68^low^ CD163^high^) macrophages was present within the areas of ^19^F signal (supplemental Figure V). The mean ^19^F-signal intensity was quantified considering the coil sensitivity profile (supplemental Figure VIII) and expressed as myocardial ^19^F-SNR. The calculated local mean ^19^F SNR acquired in vivo correlated with the mean monocyte/macrophage density determined by histology (Fig. 3C; *R*^2^ = 0.6075, *p* < 0.0001). The individual localization of the analyzed regions of interest is given in the supplemental Table V. The ^19^F detection threshold was 70,000 cells/mm^3^. In sham pigs, no myocardial ^19^F signal was observed, and immunohistochemistry did not show signs of acute myocardial inflammation.

**Fig. 4:**
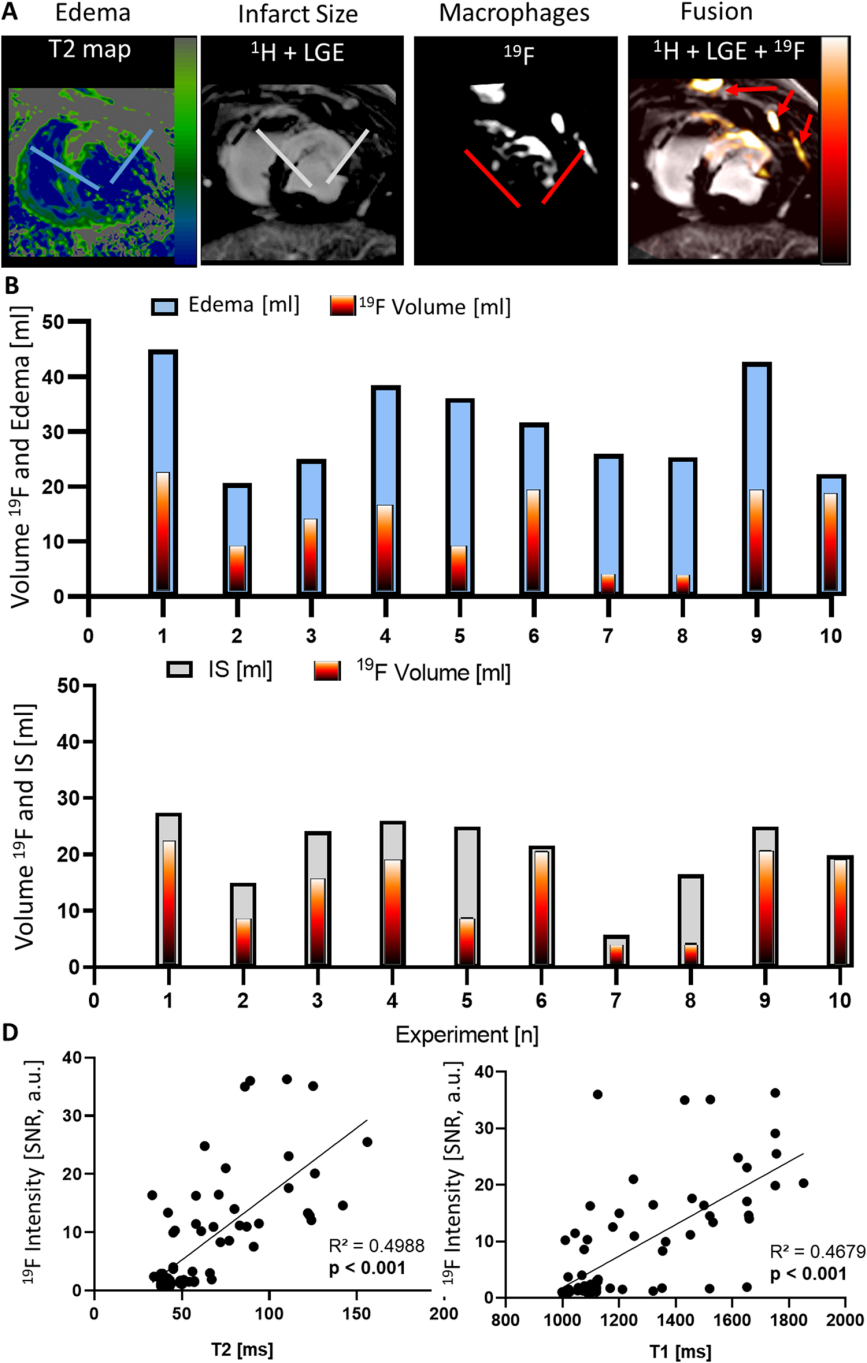
^19^F signals co-register with the infarct area. **A** ^1^H images with T2 mapping for identification of edema (blue lines) and late gadolinium enhancement (LGE) for infarct size (gray lines) were recorded (left) and fused with ^19^F images representing patchy macrophage presence (red lines). The ^19^F image overlay with the ^1^H image was performed automatically encoded with the “hot iron-lookup table” (HILT) in arbitrary units (right). Red arrows = signals in the sternum and ribs. Quantitative analysis of individual whole heart ^19^F volumes (HILT) and **B** edema extent (blue) as well as **C** infarct size (gray). **D** The ^19^F signal-to-noise ratio (SNR) of infarcted segments was significantly correlated with local T2 and T1 times.

Figure 4 shows the individual whole heart quantitative ^19^F-analysis of all surviving pigs. For inter-individual image comparison, ^19^F-signals of all 10 pigs are shown in supplemental Figure VII. The monocyte-dependent ^19^F-signals were also observed in the bone marrow of the sternum and ribs (Fig. 4A and supplemental Figure IX). These signals could easily be distinguished from AMI signals and did not disturb the signal quality or quantification. After cardiac segmentation, the average volume covered by ^19^F-signals per heart was 15.8 ± 9.5 ml, and it was always within and smaller than the respective individual edema extent (31.3 ± 8.7 ml) (Fig. 4A, B) and IS (20.9 ± 6.5 ml) (Fig. 4A, C). The mean myocardial ^19^F SNR was 13.3 ± 4.7 with a considerable inter-individual and segment-wise variation. As shown in Fig. 4D, there was a correlation of ^19^F-SNR with ^1^H derived T1 and T2 times taken from the infarcted segments of all pigs. Along with the systemic inflammatory response, we identified a correlation (*R*^2^ = 0.66) of the individual increase in circulating leukocytes from baseline until day 3 and the ^19^F integral (supplemental Figure III D).

### Segmental ^19^F- and ^1^H-CMR-derived infarct tissue characteristics

**Fig. 5:**
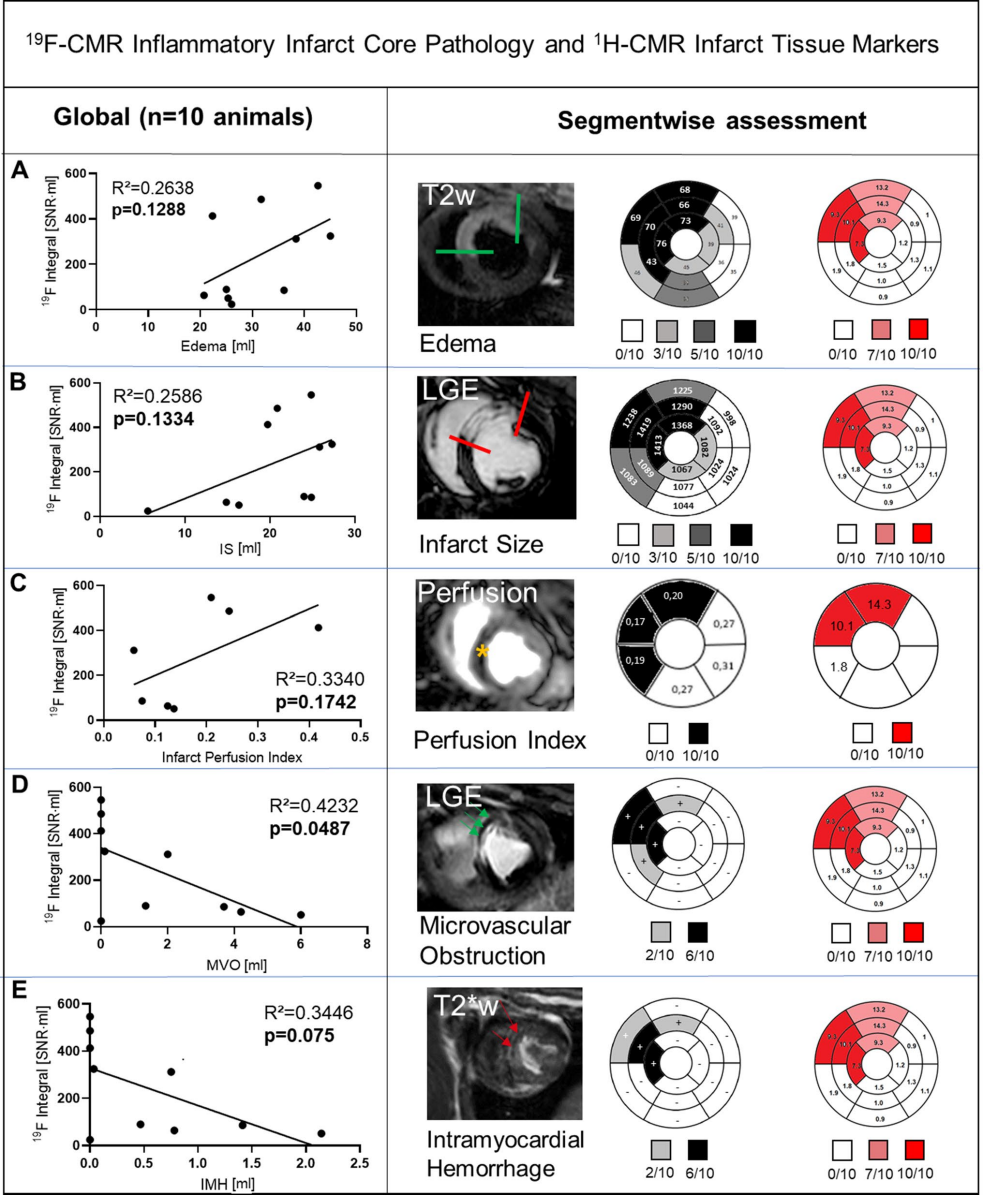
Segmental analysis of ^19^F integral and infarct tissue characteristics. Whole heart correlation of the ^19^F integral (Signal-to-noise-ratio [SNR] * ml) with infarct tissue characteristics (ml): **A** edema extent, **B** infarct size (IS), **C** regional perfusion, **D** microvascular obstruction (MVO) and **E** intramyocardial hemorrhage (IMH). The whole heart analysis, using a bivariate correlation model, revealed only MVO inversely correlated with ^19^F volume. Global and regional (segmental) analysis for the respective infarct pattern is given with example images (edema extent = green borders, IS = red borders, regional perfusion defect = yellow star, MVO = green arrows, IMH = red arrows). The bullseye plots are shown with color intensity coding according to the individuals positive for the respective segment. Additionally, ^19^F Signal to Noise Ratio, T1 and T2 values, as well as perfusion index, are given in mean values (*n* = 10 animals) per segment. IMH and MVO segments are indicated with “−”and “ + ”

As shown in Fig. 5A + B, not all myocardial segments with edema and infarction were positive for the ^19^F-CMR derived monocytes/macrophages signals. The correlation of edema extent and IS with the ^19^F-integral was only fair. Pigs with high ^19^F integral displayed only minor volume of MVO with concomitant IMH (Fig. 5 D + E). Apparently, the ^19^F-CMR derived monocytes/macrophages signal was of patchy nature with only fair correlation to edema extent or IS, and the ^19^F integral was reduced in pigs with MVO and IMH. As shown in the supplemental figure X, hemorrhage itself had no impact on ^19^F SNR.

### Association of border zone ^19^F-signal with remote myocardial contractile function and initial ventricular remodeling

**Fig. 6:**
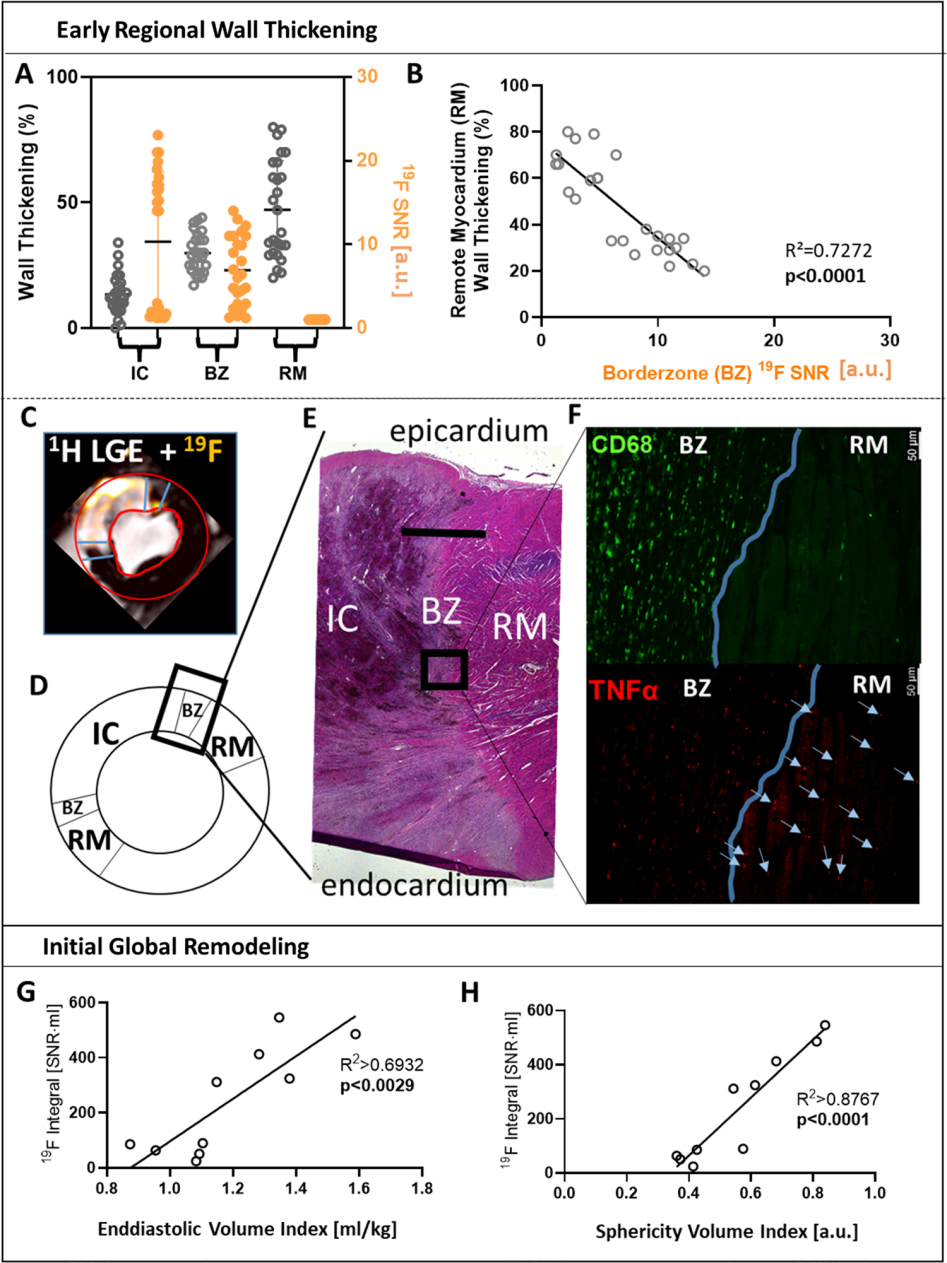
The border Zone ^19^F signal is associated with impaired remote systolic wall thickening and the whole heart ^19^F integral with initial remodeling post-AMI. **A** Myocardial systolic wall thickening (% of end diastolic thickness, gray) and ^19^F signal-to-noise ratio (SNR, orange) of the infarct core (IC), border zone (BZ) and remote myocardium (RM). **B** Correlation of RM systolic wall thickening to BZ ^19^F SNR. (C) ^19^F signal distribution across IC, BZ and RM as delineated by ^1^H magnetic resonance imaging with late gadolinium enhancement (LGE), **D** schematic assignment of left ventricular territories with region-of-interest pointing towards **E** representative histological (Hematoxylin Eosin Stain) overview of IC, BZ and RM. Scale bar 2.5 mm. **F** Histology of BZ/RM at identical region-of-interest with staining for macrophages (CD68, green) and TNF-α (red); Scale bar 50 µm. The blue line is highlighting the border of structurally intact cardiomyocytes as delineated by autofluorescence. Within BZ, there is a clear overlap of TNF-α + spots with CD68 + spots. In RM, blue arrows point towards TNF-α spots within cardiomyocytes as delineated by their autofluorescence. Correlation of whole heart ^19^F integral with end diastolic volume index (**G**) and sphericity volume index (**H**)

As shown in Fig. 6A, regional wall thickening displayed a gradient from infarct core to remote myocardium, while ^19^F SNR decreased in most pigs from infarct core to remote myocardium. Interestingly, the ^19^F-signal in the infarct border zone correlated inversely with the neighboring remote myocardial wall thickening (Fig. 6B)*.* Histological analysis revealed significant TNF-α signals in remote cardiomyocytes of those pigs with high border zone ^19^F SNR (6F + 6G). Increased ^19^F-integrals over the entire heart correlated with enlarged EDVi and ShVi in univariate analysis. Even when adjusted for edema, IS, MVO and IMH, respectively, in multivariate models, the macrophage/monocyte derived ^19^F-signal was independently associated with initial LV remodeling (supplemental Table VI).

**Fig. 7:**
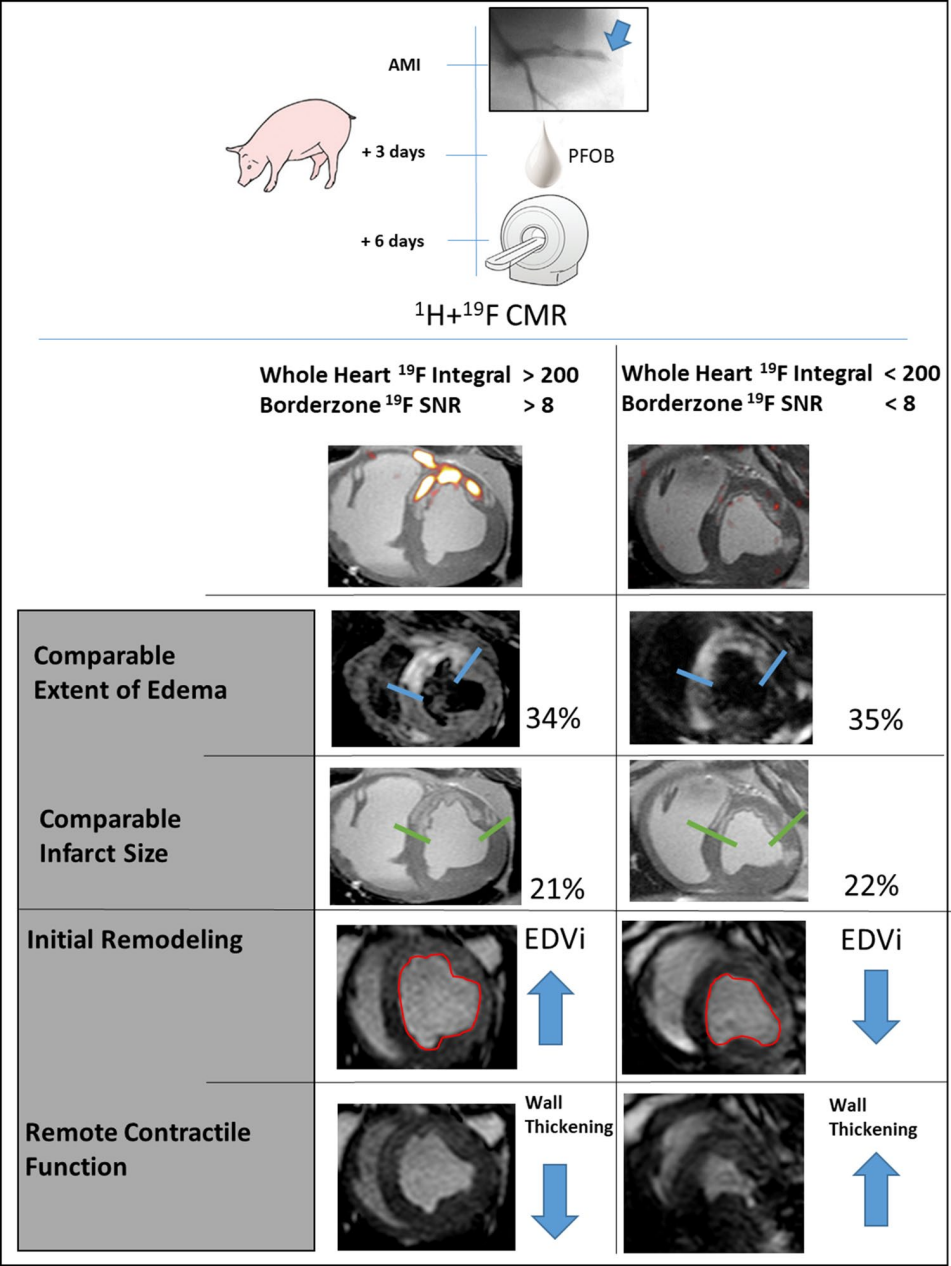
^19^F signaling provides additional information on initial remodeling and remote myocardial contractile function early after myocardial infarction. At comparable infarct sizes and areas at risk, increased macrophage signals delineated by ^19^F-CMR are associated with impaired remote myocardial contractile function and remodeling early after AMI. Representative images of two pigs after stratification for the whole heart ^19^F integral > / < 200 SNR × ml and border zone ^19^F SNR > / < 8 are shown. The edema extent (blue borders) and infarct size (green borders) were comparable. Images of LV areas in end diastole and end systole display differences in remote myocardial contractile function and ventricular shape. The ^19^F cut-off identifies pigs with impaired remote wall thickening and larger end diastolic volume index (EDVi).

As demonstrated in Fig. 7, a cut-off ^19^F-integral value of 200 combined with a border zone SNR > 8 predicted impairment of remote myocardial contractile function and LV remodeling on day 6 after AMI. Pigs with whole heart ^19^F-integral > 200 and border zone SNR > 8 (*n* = 5) had less remote myocardial systolic wall thickening (29.4 ± 6 vs. 59 ± 16%, *p* = 0.005), larger EDVi (1.4 ± 0.1 vs. 1.0 ± 0.1 ml/kg body weight, *p* = 0.005) and larger ShVi (0.7 ± 0.1 vs. 0.4 ± 0.1, *p* = 0.005) than pigs with whole heart ^19^F-integral < 200 and border zone SNR < 8 (*n* = 5).

## Discussion

In the present study using a pig model of reperfused AMI, ^19^F-CMR noninvasively provided quantitative information about regional inflammatory infiltration of monocytes/ macrophages early after AMI and its association with LV contractile function and geometry. Inflammatory infiltration in the infarct border zone correlated with impaired remote myocardial systolic wall thickening and initial LV remodeling. The inflammatory signal over the entire heart correlated to impaired LV contractile function and remodeling independently of myocardial edema, IS, MVO, and IMH.

### In vivo imaging of monocytes/macrophages with ^19^F-CMR

Our preliminary experiments were conducted to test and prove the applicability of various components and processes necessary for the planning, initiation and conduction of the present study. First, we tested optimal ^19^F containing nanoemulsions with respect to safety, dose and timing of the emulsion administration [4]. ^19^F measurements were done in explanted hearts to assure easy and sufficient ^19^F signal acquisition [4]. Thereafter, we tested and improved the in vivo CMR approach-applicability with ^19^F signal distance > factor 100 compared to explanted hearts and respiratory as well as cardiac movement [33]. After these experiments, it was clear, that a bFFE sequence with an off-set frequency of 58 ppm would be the best compromise for signal precision and SNR [33].

The findings of these preliminary studies (dose, timing, optimized sequences, work flows) were necessary to efficiently conduct the present study. The specific novelty of the present approach is the comparison of state-of-the-art CMR tissue markers as well as local and global myocardial function after AMI with the in vivo ^19^F signal, which was also validated by histology.

Since ^19^F-CMR is essentially background-free, perfluorocarbon nanoemulsions are the primary source of the ^19^F-signal, and they are preferentially taken up by monocytes/macrophages [3, 26]. In the present study, the nanoemulsion was administered on day 3 after AMI to allow for sufficient uptake into monocytes/macrophages [26] and to induce a maximum monocyte/macrophage signal in the injured myocardium on day 6 after the insult. Untargeted perfluorocarbon nanoemulsions are taken up by M1 and M2 tissue macrophages to an equal extent [13]. Since both cell types were present in equal numbers in our study on day 6 after reperfused AMI, the ^19^F-signal observed reflected most likely a mixture of a M1 and a M2 macrophage infiltration. Additionally, the ^19^F-signal was closely associated with altered tissue relaxation properties indicating early tissue reorganization in infarcted myocardium with a high volume and density of monocytes/macrophages.

As an alternative to ^19^F labeling of immune cells, ultrasmall iron oxide nanoparticles (USPIOs) enhanced ^1^H-based CMR has been used. USPIOs also preferentially label monocytes/macrophages and significantly affect ^1^H relaxation properties. This technique, leading to signal voids in the area of monocytes/macrophages, has provided initial, promising diagnostic results in patients after AMI, but its specificity has been hampered by interference with IMH [29, 46]. Beyond direct visualization of immune cells, the metabolic signature of inflammation can be assessed with positron-emission tomography (PET) or hyperpolarized CMR [23, 24, 31, 32].

PET MRI using 18F-fluorodeoxyglucose (18F-FDG) has proven significant value in the detection of atherosclerosis-related inflammation and in predicting restenosis in peripheral artery disease [5, 8]. There are specific nuclear PET tracers for imaging monocytes/macrophages after AMI [30, 31, 39]. Indeed, combined PET-MRI approaches have identified certain cell types and inflammatory activity using optimized image resolutions of 4 × 4 × 4 mm^3^ [8]. However, a direct quantification of the signal in terms of local cell abundance is difficult due to tracer/image acquisition timing and complex post-processed image reconstructions. The need for cost intensive specialized equipment (linear accelerator) and staff (chemist) might hamper the applicability of PET/MRI in clinical routine. The ^19^F principle easily works on the widely available CMR platform, with an image resolution (1.35 × 1.35.1.5 mm^3^) sufficient for imaging inflammatory foci in the AMI border zone with a directly quantifiable signal, without the need for additional specialized equipment and personnel.

With the present CMR approach, the images of monocyte/macrophage infiltration were generated in vivo with a resolution and acquisition time comparable to clinical standard sequences and protocols (supplemental Table I). The pig model of reperfused AMI in the present study exhibited a similar extent of IS and frequency of MVO or IMH as typical patients with an anterior STEMI [6, 29]. Moreover, the monocyte/macrophage density was comparable to histopathological studies in patients [42], rendering the present model suitable for translational perspectives. Thus, ^19^F-CMR is a highly promising imaging technique for clinical trials aiming to further evaluate the effects on monocyte/macrophage infiltration in patients early after AMI.

### Inflammatory infiltration and remote myocardial contractile function and initial left ventricular remodeling

In vivo myocardial ^19^F-signals revealed not only considerable inter-individual variation, which correlated to the systemic increase in leukocytes (supplemental figure IIID), but also a patchy appearance in myocardial infarction, which was validated by histology (Fig. 3). The spatial heterogeneity of monocytes/macrophage infiltration was reflected in features of the ^19^F-signal: Even those pigs with low ^19^F whole heart volumes (< 5 ml) had a mean myocardial ^19^F-SNR of > 10, indicating hot spots of monocyte/macrophage infiltration as known also from human autopsy studies [42]. The area of myocardial edema and IS was not entirely invaded by monocytes/macrophages as evidenced by ^19^F-CMR. Moreover, the incidence of MVO and IMH was even reduced in segments positive for ^19^F-CMR. Since MVO and IMH coincided in our model (day 6 after AMI), in good agreement to clinical observations [29], the attenuating effect of MVO on monocyte/magrophage infiltration, which has already been shown by other groups [45], might have counterbalanced the pro-inflammatory effect of IMH at day 6 [2].

Our present study provided novel information on the regional relationship between monocyte/macrophage infiltration in the infarct border zone with systolic wall thickening in the adjacent remote viable myocardium (Fig. 6A + B) and with LV remodeling early after AMI (Fig. 6G + H). The infarct border zone derived ^19^F-signal correlated inversely with remote myocardial systolic wall thickening. On histology, TNF-α was identified in remote cardiomyocytes (Fig. 6F). A spillover of cytokines with a negative inotropic action such as TNF-α from the border zone into the remote myocardium might thus explain the impaired wall thickening possibly mimicking stunned myocardium [17]. It is unclear to what extent such increased TNF-α levels are derived from monocytes/macrophages or the cardiomyocytes in the inflamed border zone, as shown in microembolized myocardium [7, 18].

On the level of a global analysis over the entire heart, the global ^19^F integral was associated with LV remodeling on univariate analysis. In multivariate analysis, the correlation of the global ^19^F integral was independent from edema, IS, MVO, and IMH, respectively. The greater the monocytes/macrophages infiltration, as indicated by ^19^F-CMR, the more enlarged was EDV. Our data revealed threshold values: when the ^19^F-derived monocyte/macrophage signal exceeded 200 SNR × ml over the entire heart, it was associated with LV remodeling; when the border zone ^19^F SNR exceeded 8, it was associated with impaired remote myocardial wall thickening.

### Clinical perspectives and considerations

PFOB nanoemulsions have already been evaluated in clinical phase III studies as blood substitutes with similar application doses as used in the present study [35]. They are taken up by monocytes or macrophages irrespectively of their M1/M2 polarization in a time-dependent manner, while preserving their function [13, 26], which opens the translational perspective to develop ^19^F-based CMR imaging for future clinical applications. Due to the preferential uptake of PFOB nanoemulsions, ^19^F-based monocyte/macrophage imaging is rather cell-specific and directly quantifiable, as shown in the present study, and it might be even capable of being more specific when using functionalized nanoemulsions [11]. Intravenous administration of the nanoemulsion on day 3 after AMI enabled sufficient accumulation in monocytes/macrophages for sensitive detection by ^19^F-CMR within the infarcted myocardium on day 6 and was not associated with major side effects. Thus, ^19^F-CMR is capable to deliver in vivo information about early inflammatory infiltration after AMI. Inflammation can thus be visualized and followed as a potential additional target for subacute cardioprotection after AMI—beyond the already well established concept of preservation of coronary microcirculation [16].

Inflammation is a driver of adverse LV remodeling after AMI [44]. There are large inter-individual differences with respect to monocyte/macrophage quantity and localization in post-AMI inflammation [42]. The peri-infarct border zone, as identified by the specific gadolinium contrast agent uptake kinetics, is characterized by inflammatory infiltration, involved in the pathogenesis of arrhythmias [40] and associated with mortality [20].

For patients after AMI, it is currently unclear whether local hot spots of inflammation, a patchy pattern of inflammation in the infarct border, or the overall inflammatory volume in the heart drive clinical endpoints. The ^19^F platform might add novel information in this respect, since it specifically images monocytes/macrophages and thus can also monitor specific modulation of the inflammatory response by pharmacotherapy [21, 22, 28]. Compared to techniques using radiotracers, CMR is widely available and has better spatial resolution. In the future, the nanoemulsion delivery and the imaging workflow can easily be adopted to the management of patients after AMI in the hospital.

^1^H CMR-derived tissue markers for improved prediction of cardiovascular endpoints are not always present after AMI. Major markers of mortality after AMI, such as MVO and IMH, appear in only 40% of all patients. The ^19^F signal will most probably be detectable in every singular individual myocardium post-AMI within the infarct region and border zone, thus enabling quantification of inflammatory patterns and extent. For patients after AMI, it is currently unclear whether local hot spots of inflammation, a patchy pattern of inflammation in the infarct border, or the overall inflammatory volume in the heart drive clinical endpoints. The ^19^F platform might add novel information in this respect, since it specifically images monocytes/macrophages and thus can also monitor specific modulation of the inflammatory response by pharmacotherapy [22]. However, whether or not these additional information further enhance the risk prediction after AMI needs to be tested in clinical trials.

### Study limitations

Since the present study aimed to mimic a clinical imaging scenario at day 6 with subsequent histological validation, the predictive value of the respective ^1^H- or ^19^F-derived signals on more long-term remodeling remains unclear. In mice, increased ^19^F-derived monocyte/macrophage signals predicted worsening of LV function also 28 days after AMI [3]. The time point of CMR analysis at 6 days post-AMI was chosen in line with recent expert consensus regarding the dynamic nature of infarct development and repair [19]. Our imaging study does not establish a cause-effect relationship between early inflammatory infiltration and impaired remote contractile function or LV remodeling early after reperfused AMI, but demonstrates a close association.

## Conclusion

In vivo ^19^F-CMR provides noninvasive visualization and quantification of monocytes/macrophages with reliable sensitivity and spatial resolution. In the present study using a pig model of reperfused AMI, in vivo ^19^F-CM identifed inflammatory infiltration as independent determinant of LV contractile function and geometry early after acute myocardial infarction. In a translational perspective, in vivo ^19^F-CMmight also enable loco-regional and longitudinal cell-specific CMR based imaging in AMI patients on a widely available technological platform. Thus, ^19^F-CMR, in addition to established ^1^H-based CMR, offers the potential to identify and characterize targets for novel anti-inflammatory therapeutic agents.

## Supplementary Information

Below is the link to the electronic supplementary material.

Supplementary file1 (DOCX 5114 kb)
