# Supplementary material for: Regional analysis of inflammation and contractile function in reperfused acute myocardial infarction by in vivo 19F cardiovascular magnetic resonance in pigs
Source: Basic Res Cardiol. 2022 Apr 7;117(1):21. doi: 10.1007/s00395-022-00928-5 (PMC8989832; doi:10.1007/s00395-022-00928-5)
Supplement: Supplementary file 1 — Supplementary file1 (DOCX 5114 kb) [file 395_2022_928_MOESM1_ESM.docx]

**Regional analysis of inflammation and contractile function in reperfused acute myocardial infarction by *in vivo* ^19^F cardiovascular magnetic resonance in pigs**

Bönner F^1^, Gastl M^1^, Nienhaus F^1^, Rothe M^2,3^, Jahn A^1,4^, Pfeiler S^1^, Gross U^5^, Schultheiss H-P^5^, Ibanez B^6,7,8^, Kozerke S^9^, Szendroedi J^10^ , Roden M^2, 3, 10^, Westenfeld R^1^, Schrader J^11,12^, Flögel U^11,14^, Heusch G^13^, Kelm M^1,14^

^1^ Division of Cardiology, Pulmonology and Vascular Medicine, Medical Faculty Heinrich Heine University, Düsseldorf, Germany

^2^ Institute for Clinical Diabetology, German Diabetes Center, Leibniz Center for Diabetes Research, Düsseldorf, Germany

^3^ German Center for Diabetes Research, Partner Düsseldorf, Germany

^4^ Central animal research facility, Heinrich Heine University, Düsseldorf, Germany

^5^ Institute for Cardiac Diagnostics and Therapy (IKDT), Berlin, Germany

^6^ Centro Nacional de Investigaciones Cardiovasculares Carlos III (CNIC), Madrid, Spain

^7^ IIS-Fundación Jiménez Díaz Hospital, Madrid, Spain.

^8^ CIBERCV, Madrid, Spain

^9^ Institute for Biomedical Engineering, University and ETH Zurich, Zurich, Switzerland

^10^ Division of Endocrinology and Diabetology, Medical Faculty Heinrich Heine University, Düsseldorf, Germany

^11^ Department of Molecular Cardiology, Heinrich Heine University, Düsseldorf, Germany

^12^ Experimental Cardiovascular Imaging, Department of Molecular Cardiology, Heinrich Heine University, Düsseldorf, Germany

^13^ Institute for Pathophysiology, West German Heart and Vascular Center, University of Essen Medical School, Essen, Germany.

^14^ Cardiovascular Research Institute Düsseldorf (CARID), Medical Faculty, Heinrich Heine University, Düsseldorf, Germany

DATA SUPPLEMENT

**Table of content Pages**

**Supplemental materials & methods 4**

- Experimental pig model 4
- Anesthesia, analgesia and biomonitoring 4
- Induction of acute myocardial infarction 5
- Production and quality control of perfluorooctyl bromide nanoemulsion 7
- Application of perfluorooctyl bromide nanoemulsion 8
- Cardiovascular magnetic resonance 8
- Analysis of cardiovascular magnetic resonance data sets 10
- Autopsy and organ preparation 11
- Histological protocols 13
- Analysis of histology 15

**Tables**  **16**

- Table I: Cardiovascular magnetic resonance sequence details 16
- Table II: Baseline and procedural characteristics of all pigs 17
- Table III: Circulating biomarkers of myocardial infarction 18
- Table IV: Reaction to application of perfluorooctyl bromide nanoemulsion 19
- Table V: ^19^F signal regions-of-interest for histological validation 20
- Table VI: Correlation of ^19^F Integral, left ventricular function, initial remodeling 21

**Figures 22**

- Figure I: Induction and efficacy testing of acute myocardial infarction 22
- Figure II: Characteristics of Perfluorooctyl Bromide Nanoemulsion 23
- Figure III: Circulating leukocytes and blood kinetics of perfluorooctyl bromide nanoemulsion 24
- Figure IV: Histological validation of microvascular obstruction and intramyocardial hemorrhage 25
- Figure V: Subdifferentiation of the monocyte/macrophage population 26
- Figure VI: Image quality for ventricular volumes, function, and tissue analysis 27
- Figure VII: Fused ^1^H and ^19^F image examples of all ten pigs 28
- Figure VIII: ^19^F Signal Quantification according to coil sensitivity profile 29
- Figure IX: ^19^F Signal distribution in the reticulo-endothelial system 30
- Figure X: Association of infarct tissue characteristics with indexes of initial remodeling 31
- Figure XI: Correlation of ^19^F signal intensity and perfluorooctyl bromide concentration 32

**Supplemental References 33**

**Expanded materials & methods**

**Experimental animal model**

A total of 22 adult, grown up female Aachen minipigs were used in the present study [5]. Pigs were at least 1.5 years old (1.5 years ± 3 months). They had human-like dimensions with a mean thorax girth of 120 ± 25 cm and a body weight of 67 ± 9 kg. The pigs were purchased from a local breeder and housed at the central animal facility center of the Heinrich-Heine-University, Düsseldorf, Germany. Pigs were kept in individual cages, fed with a standard chow diet, and received tap water ad libitum.

**Anesthesia, analgesia** **and biomonitoring**

Pigs were fasted overnight before surgery. While in their quarters, they were sedated with azaperone (5 mg/kg BW IM, Stresnil®) Elanco Lilly Deutschland GmbH, Bad Homburg, Germany) followed by ketamine (10 mg/kg BW IM, Ketaset Zoetis, Berlin, Germany) and atropine sulfate (0.5 mg IM, Braun, Melsungen, Germany) with an additional dose of diazepam (10 mg IM, Ratiopharm GmbH, Ulm, Germany). After 10 - 20 minutes a cannula was introduced into a superficial ear vein, and anesthesia was induced with sodium thiopental (4 mg/kg IV initially and then as needed, Rotexmedica GmbH, Trittau, Germany). Intubation of the pigs in supine position was performed using a size-matched orotracheal tube (7.5 – 8.5 mm tube). Anesthesia was maintained with a mixture of isoflurane (1.5 – 2.0 % v/v, Piramal Critical Care Deutschland GmbH, Hallbergmoos, Germany) dissolved in 100 % oxygen. Pigs were ventilated with a respirator (Sulla 808 V, Dräger, Lübeck, Germany) at a rate of 10 to 12 breaths per minute, tidal volume was 450 ml (range 400 – 550 ml). For maintenance of body temperature, the animals were positioned on a heating mat on the operating table, and rectal temperature was monitored. Biomonitoring was assured by continuous monitoring of ECG, blood pressure, oxygen saturation (Monitor Eagle 4000, Marquette Heilige GmbH, Freiburg, Germany) and intermittent analysis of arterial blood gases. Physiological saline (2 mL/kg hourly, Braun, Melsungen, Germany) was infused through the ear vein during surgery. Every 20 to 30 minutes the pigs received a bolus of fentanyl, thereby maintaining analgesia (7.5 µg, Rotexmedica GmbH, Trittau, Germany).

**Induction of acute myocardial infarction**

Acute myocardial infarction was induced using a minimally invasive "closed chest”-approach. Baseline and procedural data are given in *Table II*. Under sterile conditions, a skin incision in the pelvic area was made, and the femoral vein and artery were cannulated using 6-French (F) introducer sheats (Radiofocus ® introducer II, Terumo, Eschborn, Germany). The femoral vein was used to infuse medication and to take blood samples for laboratory analysis in the central laboratory facility of the University Hospital Düsseldorf. For prevention of ventricular fibrillation (VF), animals received amiodarone (300 mg, Ratiopharm GmbH, Ulm, Germany) dissolved in Glucosteril® 5% (250 ml, Fresenius, Bad Homburg, Germany) and 5 mg metoprolol (Carinopharm GmbH, Elze, Germany) at the beginning of the procedure. In our study the β-blocker was given with a dose 3 to 100 times less than used in clinical trials and experimental studies on cardioprotection in which metoprolol was given at the end of ischemia prior to reperfusion. Since metoprolol reduces neutrophil migration into infarcted myocardium our protocols imply a potential conservative error with attenuation of myocardial inflammation after AMI [2]. For illustration of the whole experimental work-flow and efficacy testing please see *Figure I*. All procedural steps were done under fluoroscopy (BV Pulsera 9, Philips Healthcare, Hamburg, Germany). A standard wire 260 cm, 0.65 inch (Cordis, Santa Clara, CA, USA) was inserted via the femoral artery towards the aortic valve. A 6F pigtail catheter (Cordis, Santa Clara, CA, USA) was inserted over the wire and placed in the left ventricle. The pigtail catheter was used to perform a ventriculography to assess baseline ejection fraction (EF). Baseline data from left ventriculography were analyzed off-line with Cass Left Ventricular Analysis (Pie Medical Imaging BV). Borders were drawn manually in two cine frames of 90° difference followed by automatic calculation of EF. Thereafter, a 6-F Amplatz left right 1-2 guiding catheter (Medtronic, Inc., Minneapolis, MN, USA) was inserted and advanced towards the ostium of the left coronary artery. The vascular anatomy was visualized by infusing the contrast agent Accupaque© (General Electronic Healthcare, Solingen, Germany). The ostium of the left coronary artery was intubated with sequential administration of contrast medium. Heparin (10.000 I.E. Heparin-Natrium 25000, Ratiopharm GmbH, Ulm, Germany) was injected to prevent coagulation. A pressure wire (Abbott Vascular, Santa Clara, CA, USA) was inserted into the left anterior descending (LAD) coronary artery to measure distal coronary pressure (Pd) using dedicated software (Coroflow, Coroventis, Uppsala, Sweden). Depending on the size of the coronary artery, a 2.75-3.0 x 12-15 mm diameter percutaneous transluminal coronary angioplasty (PTCA) balloon (Boston Scientific, Maple Grove, MN, USA) was inserted over the coronary guide wire and advanced into the LAD distal to its first diagonal branch. Inflation was done with 8 atmospheres using the inflator set Encore™ (Boston Scientific, Ratingen, Germany). Complete occlusion of the LAD was confirmed by repeated infusion of contrast agent, measurement of coronary wedge pressure (CWP) during ischemia and ECG changes (ST-elevation). In sham experiments (n=5), the balloon was not inflated. Ischemia was maintained for 90 minutes, and the ECG was continuously checked for ventricular arrhythmias. In case of ventricular fibrillation, defibrillation was performed (Cardio Serv, Defibrillator, Hellige, Freiburg, Germany) by applying 360 J with the paddles pressed to the anterior chest wall in *loco typico* following a precordial punch. Resuscitation attempts were terminated if unsuccessful for more than 20 minutes. After 90 minutes of ischemia, the PTCA system was released, and reperfusion of the LAD was checked by renewed infusion of contrast agent and return of CWP to baseline values of Pd. At the end of the surgery, wound closure was performed, and the pigs were allowed to recover their spontaneous breathing with subsequent extubation. Transportation back to their quarters was allowed only when arterial oxygen tension had stabilized above 95 % and ECG recordings were stable. During the following days after surgery, animals were observed at least twice daily for locomotor activity, respiratory changes, body temperature, food and water intake as well as wound healing. Analgesia was maintained during the first 72 hours after surgery by using buprenorphine (5µg/kg BW IM, Temgesic ®, Reckitt Benckiser Healthcare, Hull, UK, Germany). At day 6 after AMI, the catheter examination was repeated to document patency of the LAD. Blood sampling was performed on days 1, 3 and 6. The results of the analysis in the central laboratory facility of the University Hospital Düsseldorf are given in *Table III*.

**Production and quality control of perfluorooctyl bromide nanoemulsion**

For 400 ml Nanoemulsion (NE), a buffer consisting of 0.24 g sodium dihydrogenphosphate (NaH2PO4, Carl Roth, Karlsruhe, Germany), 2.46 g disodium hydrogenphosphate (Na2HPO4, Carl Roth, Karlsruhe, Germany), 1.15 g sodium chloride (NaCl, Carl Roth, Karlsruhe, Germany) and 284.5 g water for injection purposes was prepared. 18.4 g Lipoid E 80S (Lipoid, Ludwigshafen, Germany) and 8.62 g diblock = 1 perfluoro-n-hexyl-decane (Abcr GmbH, Karlsruhe, Germany) and 322 g perfluorooctyl bromide = PFOB (Abcr GmbH, Karlsruhe, Germany) were added to the buffer with continuous stirring. The mixture remained on the magnetic stirrer until two phases had formed. The mixture was then transferred to a microfluidizer (Microfluidics Corp. Westwood, CA, USA). The emulsion remained in the microfluidizer for ten cycles with a pressure of 1000 bar resulting in an emulsion with nanoparticles of an average particle size of 180±26 nm. Particle size was determined using photon correlation spectroscopy (PCS) on a Zetatrac (Betatek, Toronto, Canada) device. Afterwards the PFOB-NE was autoclaved (30 min at 121 °C) using a program to autoclave pure liquids. If the PFOB-NE met the requirements, it was transferred under the sterile bench and stored at 4 °C until use. Each time a new batch of PFOB-NE was produced, a volume of 5 ml was removed, for checking the quality of the batch before use and for testing the durability of the PFOB-NE. Each batch was rechecked before use. The durability tests included a visual and an analytical part. In the visual part, the solution was examined for optically recognizable changes. The analytical part included the analysis of the particle size distribution using PCS and a smear on a blood agar plate to detect possible bacterial contamination. The mean particle size was determined at the PCS and compared with the data collected immediately after production. In addition, the emulsion was checked for the presence of particles ≥ 5 µm on light microscopy. A detailed description of the quality assessment is given in the *Figure II*.

**Application of perfluorooctyl bromide nanoemulsion**

Before and after administration of PFOB-NE, blood samples were withdrawn for laboratory analysis in the central laboratory facility of the University Hospital Düsseldorf. Ten surviving AMI pigs and 5 sham animals received a body-weight-adapted amount of PFOB-NE (5 ml PFOB-NE per kg body weight) at day 3 after AMI. PFOB-NE was administered under anesthesia as described above. For this purpose, the pigs were anesthetized as described above (anesthesia and analgesia). The PFOB-NE infusion rate was 80 ml/h. During infusion (4 hours ± 31 minutes) and in the course of 24 hours following infusion, pigs were checked for any immediate side effects (tachypnoe, tachycardia, rush) and long term side effects (abnormal behavior). The results are summarized in *Table IV*. For illustration of the blood half-life of PFOB-NE please see *Figure III*.

**Cardiovascular magnetic resonance**

For cardiovascular magnetic resonance (CMR), pigs were anesthetized as described above. Anesthesia was maintained with a mixture of isoflurane (1.5 – 2.0 % v/v, Piramal Critical Care Deutschland GmbH, Hallbergmoos, Germany) dissolved in 100 % oxygen. Adequate anesthesia was monitored by testing the interclaw reflex. During CMR, heart rate was monitored. When necessary, additional anesthesia was provided by administering fentanyl (7.5µg every 30 minutes, Rotexmedica GmbH, Trittau, Germany) or ketamine (Ketaset 100 mg/ml, Zoetis, Berlin, Germany).

CMR was performed at day 6 after acute myocardial infarction using a whole-body 3.0 T Achieva X-series MR scanner (Philips Healthcare, Best, the Netherlands). *In vivo* CMR was performed according to previously established animal handling, anesthetic and CMR workflow protocols [6, 7]. For ^1^H measurements two flexible double array surface coils of 14 x 17 cm and 20 cm diameter (SENSE Flex M and SENSE Flex L surface coil, Philips Healthcare, Best, the Netherlands) were used. The CMR scan consisted of a ^1^H protocol with infarct assessment and a ^19^F protocol for macrophage imaging with tested and optimized sequences (for details please see *Table I*): LV volumes (enddiastolic volume=EDV, endsystolic volume=ESV, stroke volume=SV) and function (ejection fraction=EF, global longitudinal strain=GLS, early diastolic strain rate=SRe and wall thickening=WT) were assessed with a balanced fast gradient echo (bTFE) sequence; for analysis of the edema size a turbo spin echo (TSE) sequence with SPIR (spectral presaturation with inversion recovery) fat suppression in black blood mode was used, and intramyocardial hemorrhage (IMH) was recorded with a T2* weighted balanced turbo field echo (bTFE). For quantification of edema and infarction, T2 and T1 mapping was conducted using a GRaSE (GRadient And Spin Echo) and a MOLLI (MOdified Look Locker Imaging) sequence with a ‘3(3)5’ inversion scheme. Myocardial first pass perfusion imaging was performed with a bTFE sequence while injecting a bolus of 0.1 mmol/kg body weight Gadoteridol (Pro Hance®, 0.5 M, Bracco Imaging Deutschland GmbH, Konstanz, Germany). Late gadolinium enhanced imaging (LGE) was performed 10 and 15 min after bolus injection of 0.2 mmol/kg [6]. For LGE-based infarct size measurements (at 10 minutes) and assessment of microvascular obstruction=MVO (at 15min) a fast gradient echo sequence was used. After acquisition of ^1^H reference scans, the pigs were removed from the magnet bore without losing the isocenter information, and the ^19^F coil was placed on the chest between sternum and the left leg. Thereafter, pigs were repositioned into the scanner at the same isocenter position, which was confirmed by repeating ^1^H reference scans. ^19^F imaging was performed using a balanced Fast Field Echo (bFFE) sequence with steady state free precession read-out, as described in a previous applicability study [7].

After *in vivo* CMR scans were accomplished, pigs were sacrificed inside the scanner with potassium chloride and an overdose of pentobarbital (Narcoren, Boehringer Ingelheim, Ingelheim am Rhein, Germany). Heparin (10.000 I.E. Heparin-Natrium 25000, Ratiopharm GmbH, Ulm, Germany) was injected to prevent coagulation. Thereafter, *in situ* scans were made including a high resolution ^1^H 3D Scan and the same ^19^F protocol as above. These in situ acquisitions were performed to exclude signal loss or signal spill-over due to cardiac movements and different image resolutions.

**Autopsy und organ preparation**

After *in situ* CMR scans, animals were retracted from the scanner, and an autopsy was made. Except for the infarcted heart no abnormalities could be found. Heart, spleen, liver, sternum including ribs and bone marrow as well as the vascular access site wounds were excised and stored in 4 % paraformaldehyde (PFA). After incubation for 7 days, the heart, spleen, liver, sternum including ribs and bone marrow as well as the vascular access site wounds underwent 3D ^19^F and 3D ^1^H scans. This was done with identical resolution (each with 1x1x1 mm³ isotropic resolution) to account and test for significant partial volume effects due to different image resolutions *in vivo* and *in situ*. Results of the scans of spleen, liver and bone marrow are shown in *Figure IX*. Hearts were cut in short axis slices with a medium thickness of 10 mm and again underwent e*x vivo* ^19^F scans. Here, landmarks for the identification of specific regions (right ventricular insertion points, papillary muscles etc.) were recorded for further histological processing and analysis. The heart slices were then stored in 4 % PFA for additional 2 weeks. After complete PFA-fixation of the slices, they were cut in 30 x 30 mm continuous quadrants and embedded in paraffin for further histological processing. Slices of 5 µm thickness were cut with a microtome (Jung Biocut 2035, Mikrotom, Leica Instruments GmbH, Nussloch, Germany).

**Analysis of cardiovascular magnetic resonance data sets**

*1H*

Dedicated software (Circle CVI 42, Circle Cardiovascular Imaging Inc., Calgary, AB, Canada) was used for fully automatic delineation of ventricular borders and automatic calculation of LV volumes (EDV, ESV, SV) as well as further functional parameters (EF, cardiac output, GLS, SRe, WT). As requested in the SCMR consensus document on standardized image interpretation, automatic contour delineation algorithms must be checked for appropriateness by the respective reader [8]. The automatically generated contours were thus reviewed and manually corrected, if needed. To account for inter-observer variability, the corrections were made in a two-step approach. First, a senior CMR operator (2 years of clinical and experimental CMR experience, ESC level II) corrected the contours. After that, an advanced CMR operator (10 years of clinical and experimental CMR experience, ESC Level III) validated the corrections, and corrections were fixed, when unanimous consensus was reached. The two CMR operators were blinded to group assignments of animals at the time of CMR analysis. Such manual correction of endocardial contours was necessary in 3/10 pigs after AMI.

GLS was calculated automatically by the software using the cine images and the feature tracking algorithm provided by Circle CVI 42 (Circle Cardiovascular Imaging Inc., Calgary, AB, Canada). LV volumes and cardiac output were then indexed to the body weight (ml/kg body weight) to measure enddiastolic volume index (EDVi), end systolic volume index (ESVi), stroke volume index (SVi), and cardiac index (CI, with L/min/kg body weight). Image examples for quality documentation are given in *Figure V*. Infarct size was quantified from LGE data sets using the 5-SD threshold method [6]. The myocardial border zone was characterized in line with the peri-infarct zone between 2-SD and 5-SD [3]. Myocardial edema was quantified from T2 weighted data sets using the 2-SD threshold method using entire transmural extent of edema regardless of intramyocardial signal voids due to hemorrhage and expressed as % of LV mass or in absolute volume (ml) [6]. IMH was quantified by delineating dark areas in T2* weighted images in infarct regions, and they were considered hemorrhagic, if signal void was greater than 2 standard deviations of remote myocardium, as described previously [4, 6]. MVO was quantified by manually delineating the contrast-free zones on LGE images 15 minutes after contrast agent infusion in all slices [6]. Myocardial perfusion index was calculated automatically by referencing the myocardial upslope of the signal intensity time curve against the arterial input function (ventricular SI-time curve). A sphericity volume index was calculated according to the published formula EDV/ ((π/6) * L)³ [1].

*^19^F*

Volumes containing ^19^F signals were quantified using the 3D visualization software Amira 4.0 (Mercury Systems, USA). For fusion of ^1^H/^19^F data sets HOROS (Nimble Co LLC, USA) was used. The fused images of all 10 pigs which were included in the final analysis are shown in the *Figure VII*. For quantification of the signal intensity, the primary signal at a respective region was corrected according to the coil sensitivity profile as outlined in *Figure VI*. The signal-to-noise-ratio (SNR) of respective regions was calculated from the ratio of the signal intensity mean of a region of interest (ROI) and the standard deviation of a background-noise ROI in the same slice located out of the thorax at comparable coil distance. ^19^F volumes were calculated by applying background subtraction with SNR 7 and exclusion of extracardial and right ventricular signals. The SNR of 7, a robust SNR for image interpretation, was sufficient to subtract all unspecific technical background signals (outside the body). Since technical systems (coil, scanner, software) were the same in all scans, this allowed for a consistent interindividual image interpretation. To exclude effects of blood (iron susceptibility artefacts) on the ^19^F signal, experiments with PFOB-NE in the absence or presence of blood were conducted. Here, an equal linear relationship of PFOB concentration and ^19^F signal intensity was documented regardless of the presence of blood. For details please see *Figure VIII*.

**Histological protocols**

The H.E. staining was carried out according to the following protocol:

Paraffin slices (5 µm) were incubated at 60 °C for 60 minutes in a warming cabinet. Slices were dewaxed in xylene (Carl Roth, Karlsruhe, Germany) and subjected to a series of alcohols with descending concentration to rehydrate the tissue. Incubation in hematoxylin solution (Merck KGaA, Darmstadt, Germany) for 2 minutes followed, and slices were rinsed in tap water, followed by 10 seconds color differentiation in 0.5 % hydrochloric acid solution (PanReacAppliChem, Darmstadt, Germany) and rinsed in tap water for one minute for color enhancement. Slices were then incubated in eosin solution (Carl Roth, Karlsruhe, Germany) for one minute. Slices were then subjected to an alcohol series with ascending concentration and incubated in xylene.

The Gomori Trichrome Staining was carried out according to the following protocol:

A solution was prepared by dissolving 0.6 g of Chromotrope 2R (Sigma Aldrich, Munich, Germany), 0.3 g of aniline blue (Sigma Aldrich, Munich Germany), 1 ml of glacial acetic acid, and 0.8 g of phosphotungstic acid (Merck KGaA, Darmstadt, Germany) in 100 ml of distilled water. Paraffin slices were incubated at 60 °C for 60 minutes in a warming cabinet. Slices were dewaxed in xylene (Carl Roth, Karlsruhe, Germany) and subjected to a series of alcohols with descending concentration to rehydrate the tissue. Slices were incubated for 15 minutes at 58 °C in Bouin's solution (Sigma Aldrich, Munich) and rinsed for 5 minutes under running tap water. Slices were then incubated 1:1 in Weigerts Iron Hematoxylin A + B (Carl Roth, Karlsruhe, Germany) for 5 minutes and rinsed for another 5 minutes under running tap water. Then slices were incubated for 25 minutes in the Gomori color mixture and briefly rinsed in tap water, then incubated in 0.5 % acetic acid (PanReacAppliChem, Darmstadt, Germany) for 2 minutes and subjected to an alcohol series with ascending concentration and incubated in xylene.

Immunohistochemistry for the detection of monocytes/macrophages was carried out according to the following protocol:

Tissue sections were incubated overnight at 37 °C in a warming cabinet, rehydrated and subjected to a descending series of alcohols to rehydrate the tissue. Sections were treated in a steam oven at 100 °C for 30 minutes. The sections were then incubated in citrate buffer with pH 9, which resulted in an antigen unmasking. Slices were washed with cold tap water and then incubated in PBS for 5 minutes. Slices were then incubated for 20 minutes in diluted hydrogen peroxide. Slices were washed for 5 minutes in PBS and incubated with anti-CD163 (Clone 2A10/11, ouse anti Pig, Bio-Rad Laboratories, Hercules, USA) for 60 minutes followed by a washing step in PBS. Thereafter, slices were incubated with Dako REAL EnVision Detection System (DAKO, Denmark) for 30 minutes followed by another washing step in PBS. Slices were counterstained with a carbazole containing solution for 8-10 minutes and washed in PBS. Then nuclear staining with hematoxylin (Gill II) for one 1-3 minutes was performed. Slices were washed in tap water and covered with Aquatex (Merck Millipore, Burlington, Massachusetts, USA).

Immunofluorescence staining for the differentiation of macrophages was carried out according to the following protocol. The paraffin embedded sections were dewaxed with Roticlear® (Carl Roth, Karlsruhe, Germany) and rehydrated in a decreasing ethanol concentration sequence (100%, 96%, 70%, 50%). After a washing step in Aqua dest., a heat mediated antigen retrieval using citrate buffer was performed. Unspecific binding was blocked with a blocking buffer (0.2% Fish Skin Gelatine, 0.5% BSA, 0.1% Saponin in PBS) for 1 hour. The tissue was washed in PBS and incubated with the anti-CD163 antibody (clone EDHu-1, 7.5µg/ml, #NB110-40686, Novus biological, Minneapolis, USA) over night. A secondary anti-mouse-Alexa Fluor 660 (#A-21055, 2µg/ml, ThermoFisher Scientific, Waltham, USA) antibody was used for 1 hour. After additional washing steps, the sections were incubated with anti-CD68 antibody (ab125212, 7.5µg/ml, abcam, Cambridge, UK) antibody for 10 hours. A secondary anti-rabbit-Alexa Fluor 594 (A-21207, 2µg/ml, ThermoFisher Scientific, Waltham, USA) was used for 1 hour. Alternatively, for TNF-α staining the anti-TNF-α antibody Monoclonal Mouse IgG_1_ Clone # 103302 (R&D Systems, Minneapolis USA) was used. The sections were washed in PBS and unwanted autofluorescence was diminished using the Vector® TrueVIEW® Autofluorescence Quenching Kit (Vector Laboratories, Burlingame, USA) for 2 min. The sections were mounted with Prolong Diamond with DAPI (ThermoFisher Scientific, Waltham, USA), and images were acquired using a fluorescence microscope (DM6B, Leica, Wetzlar, Germany). Stained macrophages and TNF- α spots were counted in 5-10 consecutive images of the infarct area per pig.

**Analysis of histology**

The histological examination of myocardial tissue was performed using 10x, 20x, and for more detailed images 40 x magnifying lenses (DM 4000 M microscope, Leica Microsystems GmbH, Wetzlar, Germany). At 20 x magnification, 3 adjacent infarct sections (epicardial, midmyocardial, epicardial) at 3 levels (apex, midventricular, basis) were selected randomly per pig. Macrophage count: The number of anti-macrophages antibody positive cells was quantified automatically with a software-based intensity threshold algorithm (ImageJ, NIH, freeware) and expressed as cells per mm^3^, as calculated from known in-plane resolutions of histological images at 20x magnification and slice thickness of 5µm. Intramyocardial hemorrhage: Tissue destruction and extravasation of multiple erythrocytes was assessed semi-quantitatively (0=none, 1=sparse, 2=intermediate, 3=plenty). A myocardial segment was considered positive if hemorrhage occurred with at least intermediate grading. Microvascular obstruction: In each section, the number of (micro) vessels occluded by thrombi in HE stained sections was counted and assessed semi-quantitatively (0=none, 1=sparse, 2=intermediate, 3=plenty). A myocardial segment was considered positive if microvascular obstruction occurred with at least intermediate grading. The histological results served for validation of CMR measurements of MVO and IMH. For details please see *Figure IV*.

| Sequence | Geometry | Acquisition resolution[mm] | Reconstruction resolution[mm] | TR/TE[ms] | Flip angle ° | TFE factor | Scan duration | comments |
| --- | --- | --- | --- | --- | --- | --- | --- | --- |
| Survey |  | 1.76 x 3.52 x 15 | 1.76 x 1.76 x 15 | 8/2.3 | 15° | 128 | 0:18 |  |
| bTFE | Plan 1 | 2.02 x 1.70 x 8 | 0.59 x 0.59 x 8 | 3.5/1.75 | 45° | 9 | 0:11 |  |
| bTFE | Plan 2 | 2.02 x 1.70 x 8 | 0.59 x 0.59 x 8 | 3.5/1.75 | 45° | 9 | 0:11 |  |
| bTFE | SA | 2 x 1.65 x 8 | 0.95 x 0.95 x 8 | 3.5/1.75 | 45° | 9 | 0:29 |  |
| bTFE | 2Ch | 2.02 x 1.70 x 8 | 0.59 x 0.59 x 8 | 3.5/1.75 | 45° | 9 | 0:11 |  |
| bTFE | 3Ch | 2.02 x 1.70 x 8 | 0.59 x 0.59 x 8 | 3.5/1.75 | 45° | 9 | 0:11 |  |
| bTFE | 4Ch | 2.02 x 1.70 x 8 | 0.59 x 0.59 x 8 | 3.5/1.75 | 45° | 9 | 0:11 |  |
| bTFE T2* | SA | 2 x 1.6 x 8 | 1.25 x 1.25 x 8 | 3.3/1.64 | 45° | 13 | 0:09 |  |
| T2 STIR | SA | 1.56 x 2.12 x 8 | 0.98 x 0.98 x 8 | 1500/75 | 90° | TSE factor 24 | 1:24 | Black Blood |
| T2 STIR | 2Ch | 1.56 x 2.12 x 8 | 0.98 x 0.98 x 8 | 1500/75 | 90° | TSE factor 24 | 1:24 | Black Blood |
| T2 STIR | 3Ch | 1.56 x 2.12 x 8 | 0.98 x 0.98 x 8 | 1500/75 | 90° | TSE factor 24 | 1:24 | Black Blood |
| T2 STIR | 4Ch | 1.56 x 2.12 x 8 | 0.98 x 0.98 x 8 | 1500/75 | 90° | TSE factor 24 | 1:24 | Black Blood |
| GRASE | SA | 2 x 2 x 8 | 0.5 x 0.5 x 8 | 674/X | 90° |  | 1:10 | 10 TE´s with 10 ms space |
| MOLLI | SA | 1.97 x 2 x 10 | 1.17 x 1.17 x 10 | 2.2/0.99 | 20° |  | 0:11 | Minimal TI=120 ms, ‘3(3)5’ |
| Perfusion | SA | 2.84 x 2.92 x 20 | 0.9 x 0.9 x 20 | 1.89/0.95 | 50° | 38 | 0:50 |  |
| LGE | SA | 1.37 x 1.43 x 10 | 1.4 x 1.4 x 10 | 3.571.73 | 15° | 45 | 0:11 |  |
| LGE | 2Ch | 1.37 x 1.43 x 10 | 1.4 x 1.4 x 10 | 3.571.73 | 15° | 45 | 0:11 |  |
| LGE | 3Ch | 1.37 x 1.43 x 10 | 1.4 x 1.4 x 10 | 3.571.73 | 15° | 45 | 0:11 |  |
| LGE | 4Ch | 1.37 x 1.43 x 10 | 1.4 x 1.4 x 10 | 3.571.73 | 15° | 45 | 0:11 |  |
| Survey |  | 2.34 x 4.69 x 10 | 1.76 x 1.76 x 10 | 4/1.82 | 20° | 96 | 0:40 |  |
| ^19^F bFFE | SA | 3 x 3 x 3 | 1.35 x 1.35 x 1.5 | 2.2/0.7 | 20° |  | 18:28 |  |
| T1w SE | SA | 0.9 x 1.12 x 4 | 0.5 x 0.5 x 4 | 600/10 | 70° |  | 9:25 |  |

**TABLES**

**Table I:** **Cardiovascular magnetic resonance sequence details**

Detailed table of cardiovascular magnetic resonance sequences and acquisition parameters during the *in vivo* experiments.


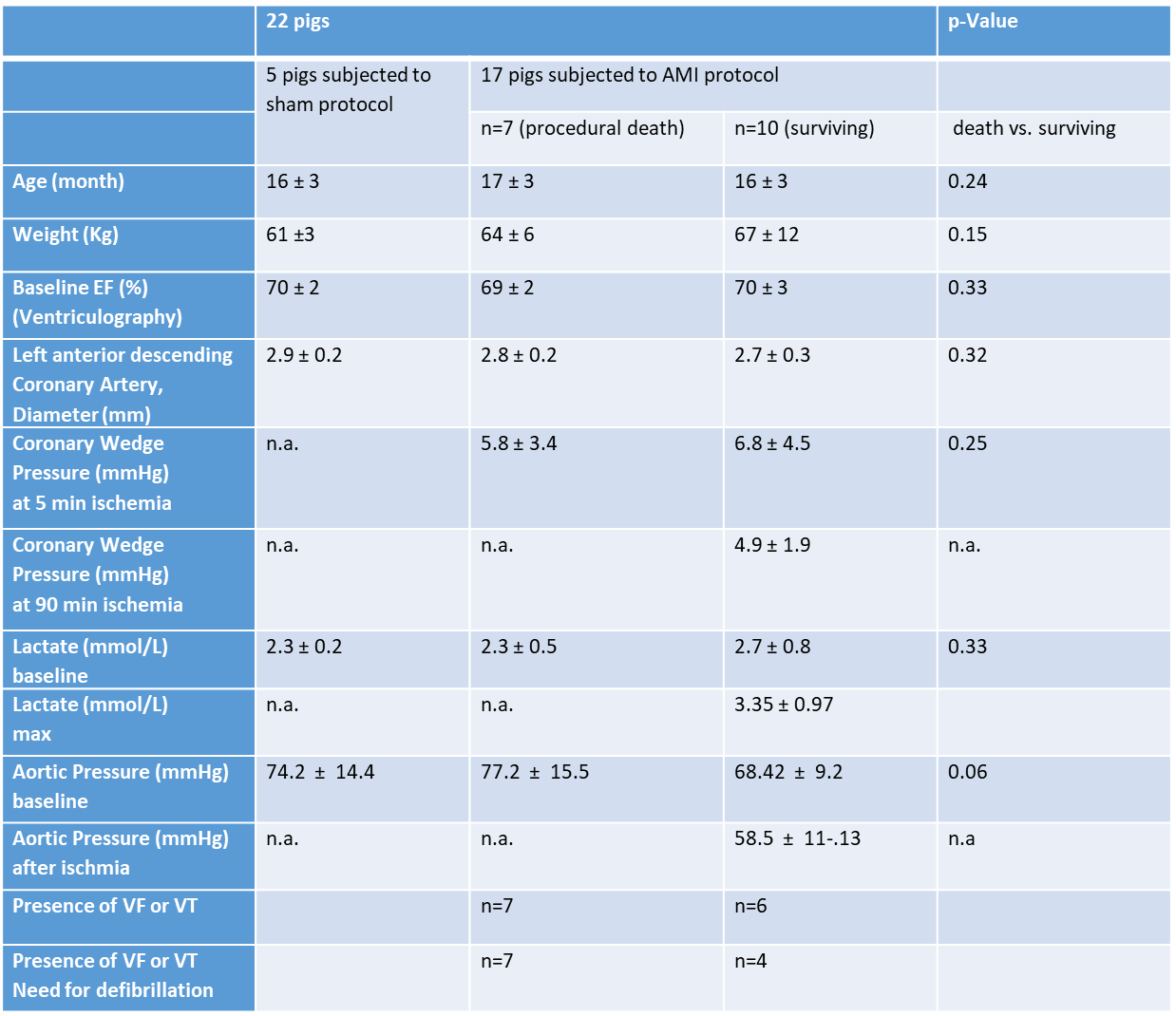


**Table II:** **Baseline and procedural characteristics of all animals**

Shown are the main anatomical, functional, and procedural characteristics of all 22 pigs. Five animals were sham operated, 17 animals were subjected to the acute myocardial infarction (AMI) protocol. 7/17 animals died during infarction from ventricular fibrillation (VF) and were analyzed separately. Shown are mean values ± standard deviation. Comparisons were performed between surviving and non-surviving AMI animals. *P*-values were calculated by unpaired student’s t-tests.


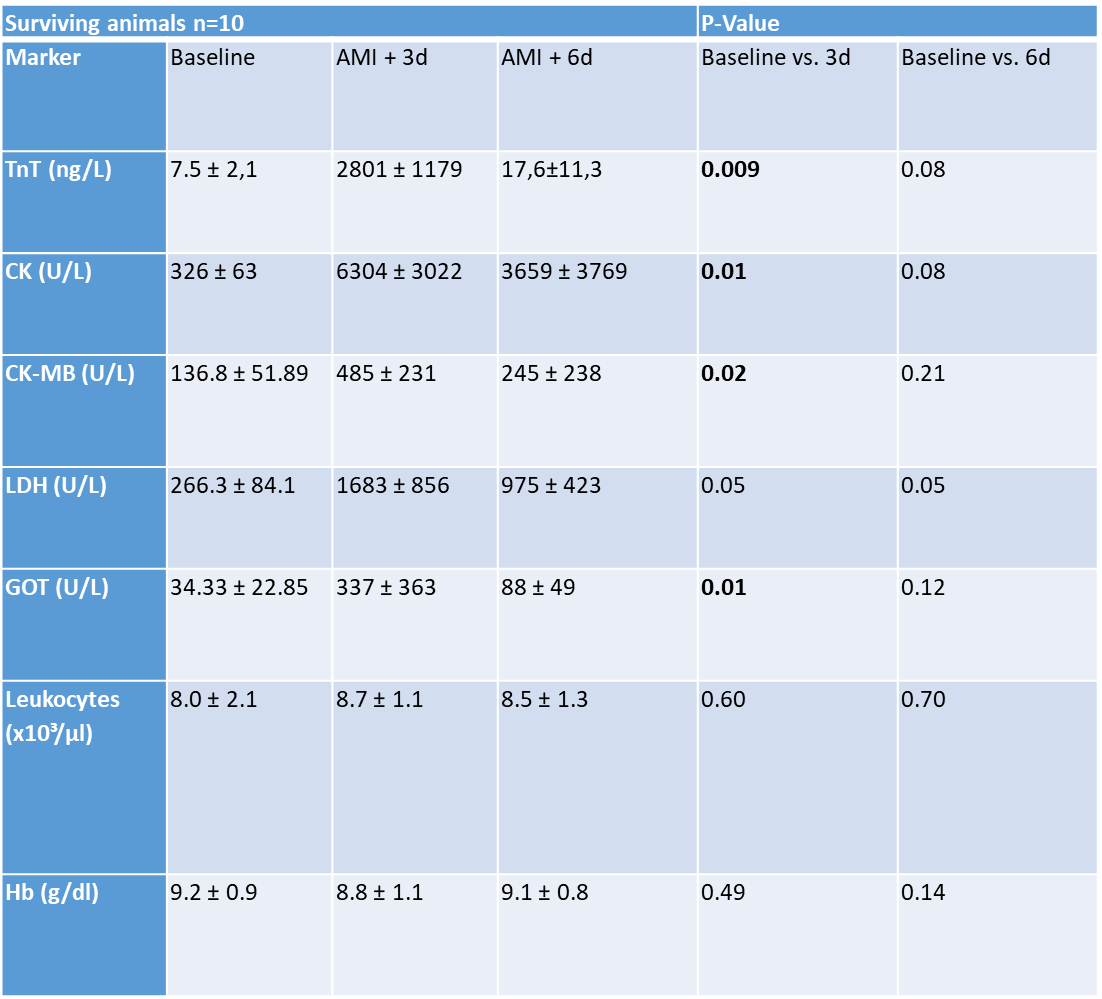


**Table III:** **Circulating biomarkers of myocardial infarction**

Shown are the circulating biomarkers troponinT (TNT), creatine kinase (CK), myocardial creatine kinase (CK-MB), lactate dehydrogenase (LDH), glutamate oxaloacetate transaminase (GOT), white blood cell count (leukocytes) and hemoglobin (Hb) of all pigs that survived the acute myocardial infarction (AMI). Analyzed time points were baseline, 3 and 6 days after AMI. Shown are mean values ± standard deviation. *P*-values were calculated by One-Way ANOVA for repeated measures and Bonferroni post-hoc tests.

| Grade 1  Grade 2  Grade 3 | *Tachypnoe*  >20/min  >25/min  >30/min | *Rushing*  During infusion  Lasting <1 h  Lasting >1 h | *Tachycardia*  >100/min  >120/min  >140/min | *Abnormal Behavior* |
| --- | --- | --- | --- | --- |
| Grade 1 | 2/15 | 2/15 | 2/15 | 0/15 |
| Grade 2 | 2/15 | 1/15 | 2/15 | 0/15 |
| Grade 3 | 4/15 | 0/15 | 4/15 | 0/15 |

**Table IV:** **Reactions to infusion of perfluorooctyl bromide nanoemulsion**

Fifteen pigs received the body weight-adapted infusion of the perfluorooctyl bromide nanoemulsion (10 with acute myocardial infarction and 5 with sham intervention). Shown are the numbers of animals with the respective reaction.

|  | *Basal Slice* | *Midventricular Slice* | *Apikal Slice* |
| --- | --- | --- | --- |
| *Animal # 1* | *3* | *2* | *2* |
| *Animal # 2* | *3* | *3* |  |
| *Animal # 3* | *3* | *2* |  |
| *Animal # 4* | *3* | *2* |  |
| *Animal # 5* | *3* | *3* |  |
| *Animal # 6* | *2* | *3* | *2* |
| *Animal # 7* | *3* | *2* |  |
| *Animal # 8* | *3* | *2* |  |
| *Animal # 9* | *3* | *3* | *1* |
| *Animal # 10* | *3* | *3* | *1* |

**Table VI. ^19^F signal regions-of-interest for histological validation**

Shown are numbers and slice location of ^19^F signal regions of interest for validation by histology for each animal.

|  | **LV function and geometry** | | | |
| --- | --- | --- | --- | --- |
|  | LV-EF | WT | EDVi | ShVi |
| **^19^F Integral, adjusted for:** |  |  |  |  |
| **Edema** | R²=.375, p=.060 | R²=.424, p=.051 | R²=.570, p=.008 | R²=.684, p<.001 |
| **IS (g)** | R²=.335, p=.072 | R²=.339, p=.079 | R²=.653, p=.005 | R²=.644, p=.001 |
| **MVO (g)** | R²=.782, p=.002 | R²=.871, p<.001 | R²=.260, p=.035 | R²=.354, p=.001 |
| **IMH (g)** | R²=.452, p=.039 | R²=.606, p=.013 | R²=.381, p=.019 | R²=.491, p=.001 |

**Table V: Adjusted p and R² values for multivariate linear regression of ^19^F integral with early left ventricular Function and initial remodeling**

Due to the limited number of observations, separate linear regression models in pairwise combination with edema, IS, MVO, and IMH were created to adjust the univariate correlations of ^19^F with LV function and volumes for these parameters. Given are adjusted p and R² values (as relative R² change tested against the full model) of the ^19^F integral within the multivariate linear regression analyses.

**FIGURES**


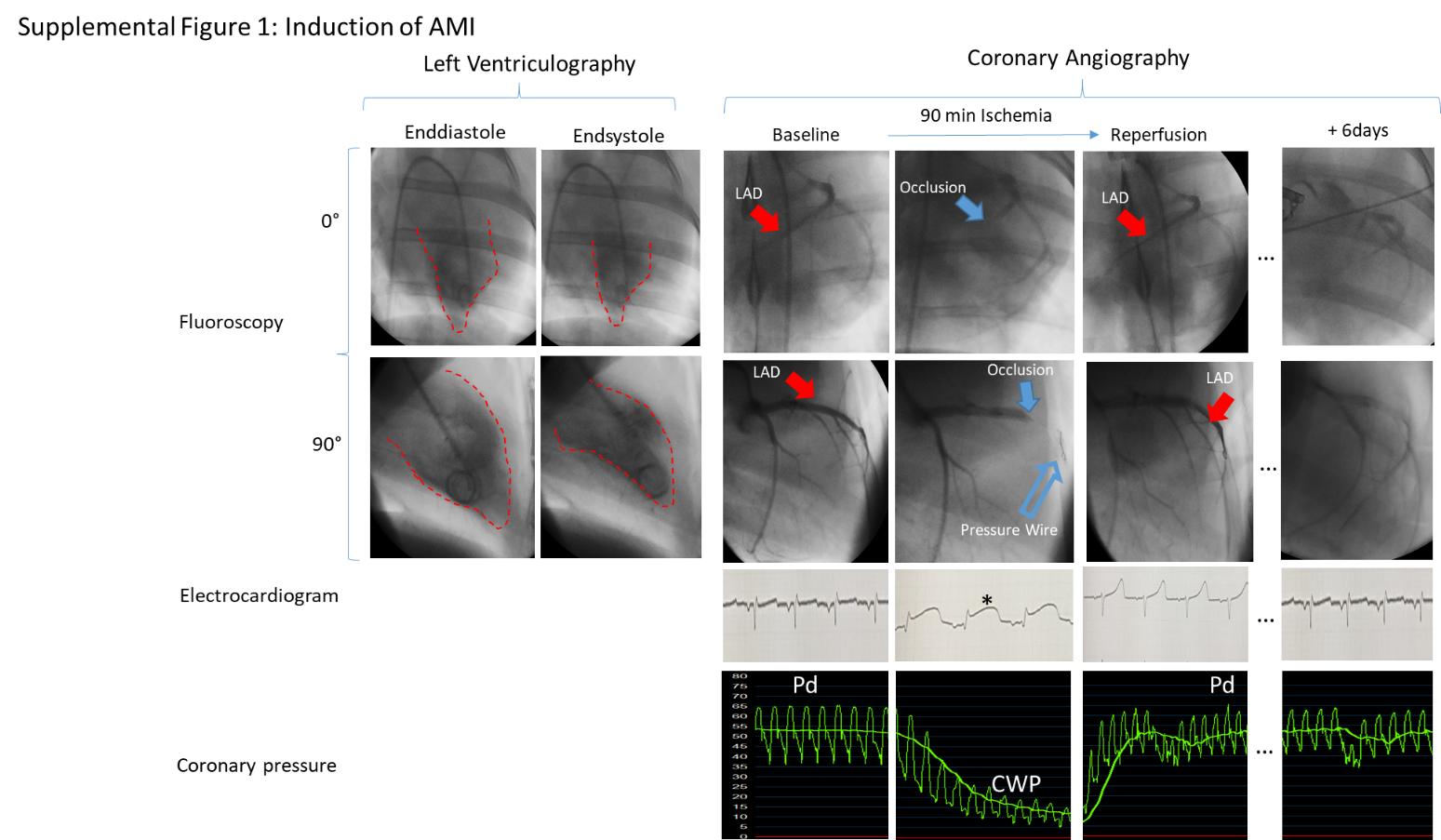


**Figure I:** **Induction and efficacy testing of acute myocardial infarction**

(Upper panel) Biplane left ventriculography and coronary angiography are displayed in 0° (above) and 90° (below). After assessment of baseline ventricular function, a first angiogram documented the patency of the left coronary artery for quantitative coronary angiography, and a pressure wire was advanced to the distal end of the left anterior descending coronary artery (LAD). Proper balloon inflation for induction of myocardial ischemia was documented by no flow in 0° (above) and 90° (below), ST-Segment elevation (*) on the ECG, and decrease of the distal coronary pressure (Pd) to coronary wedge pressure (CWP). After 90 minutes, the occlusion was released and proper patency of the LAD documented by fluoroscopy, ECG and distal Pd.


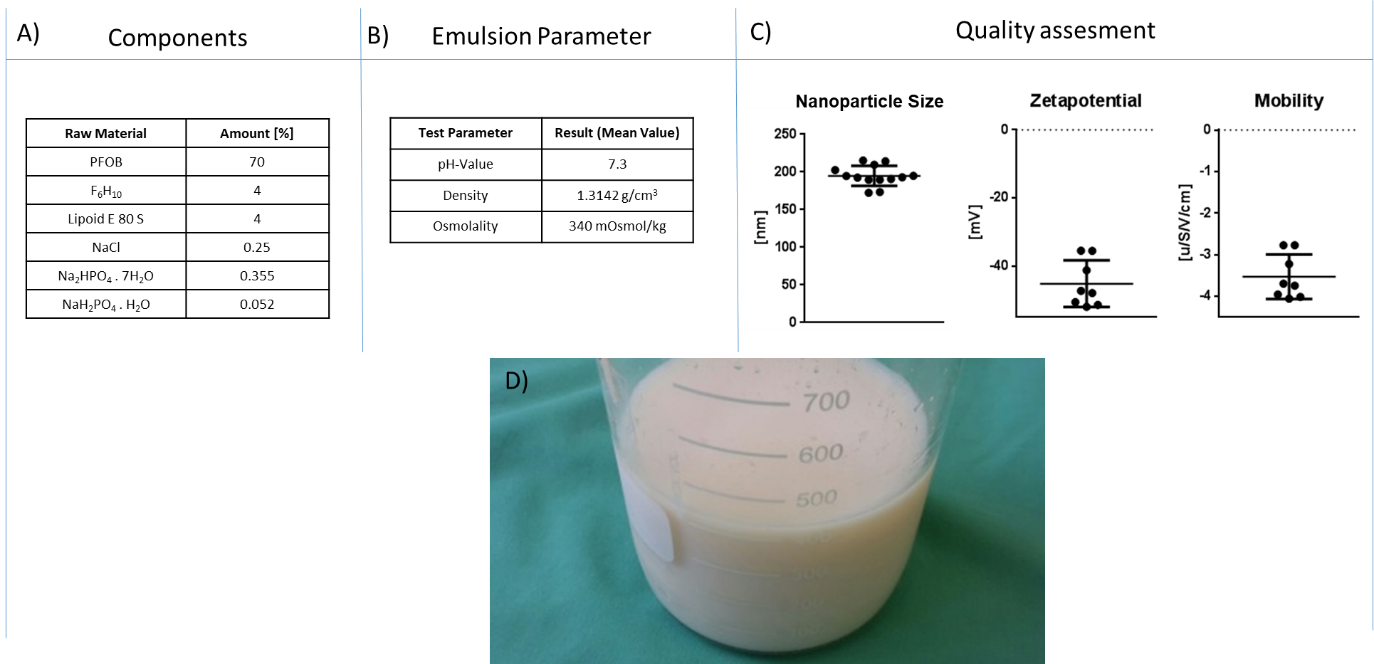


**Figure II:** **Characteristics of perfluorooctyl bromide nanoemulsion**

(A) The nanoemulsion consisted of a perfluorocarbon (perfluorooctyl bromide, PFOB), water, a phosphorous buffer system, lipoid and stabilizers (F_6_H_10_). (B) Emulsion parameters with respect to pH-value, density and osmolarity. (C) Quality assessment with respect to nanoparticle size (173 – 225 nm), Zetapotential (-38 up to -48 mV) and mobility (-2.8 up to -4.1 U/S/V/cm) of all batches used. Shown are representative batches used in the study with mean value and 95% confidence interval. (D) A typical amount of PFOB-nanoemulsion after autoclave treatment, ready for application.


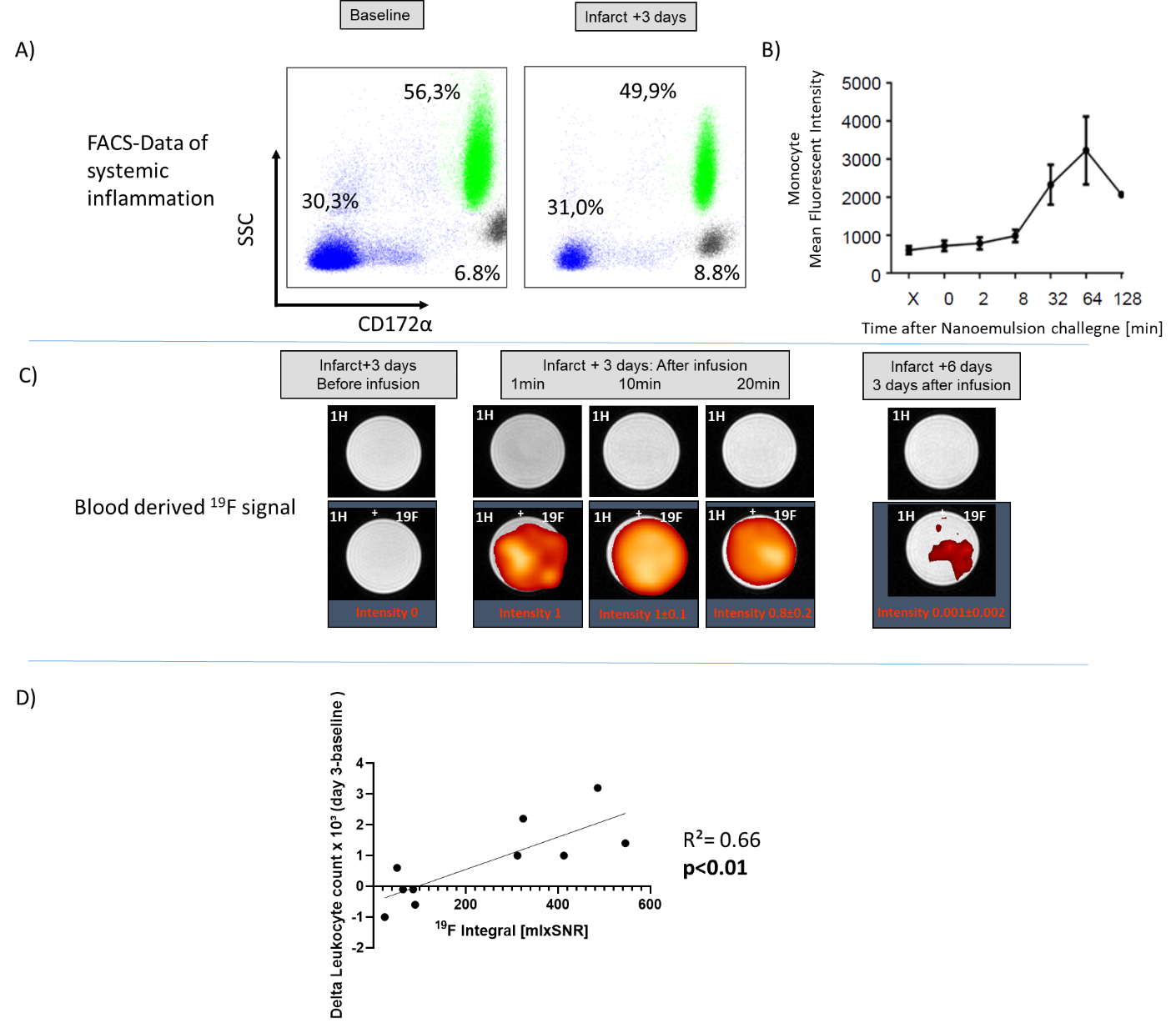


**Figure III:** **Circulating leukocytes and blood kinetics of perfluorooctyl bromide nanoemulsion**

(A) Representative fluorescence activated cell sorting (FACS) plot, 3 days after acute myocardial infarction. The relative monocyte frequency (grey) increased as compared to lymphocytes (blue) and granulocytes (green). (B) Phagocytosis assay with fluorescein labeled Nanoemulsion in whole blood. (C) Blood derived ^19^F signal intensity before and 1, 10 and 20 minutes after infusion as well as 3 days thereafter. The ^19^F signal intensity of 6 experiments is given in arbitrary units at the image bottoms as mean value with standard deviation. (D) Correlation analysis of myocardial ^19^F integral and increase in circulating leukocyte count.


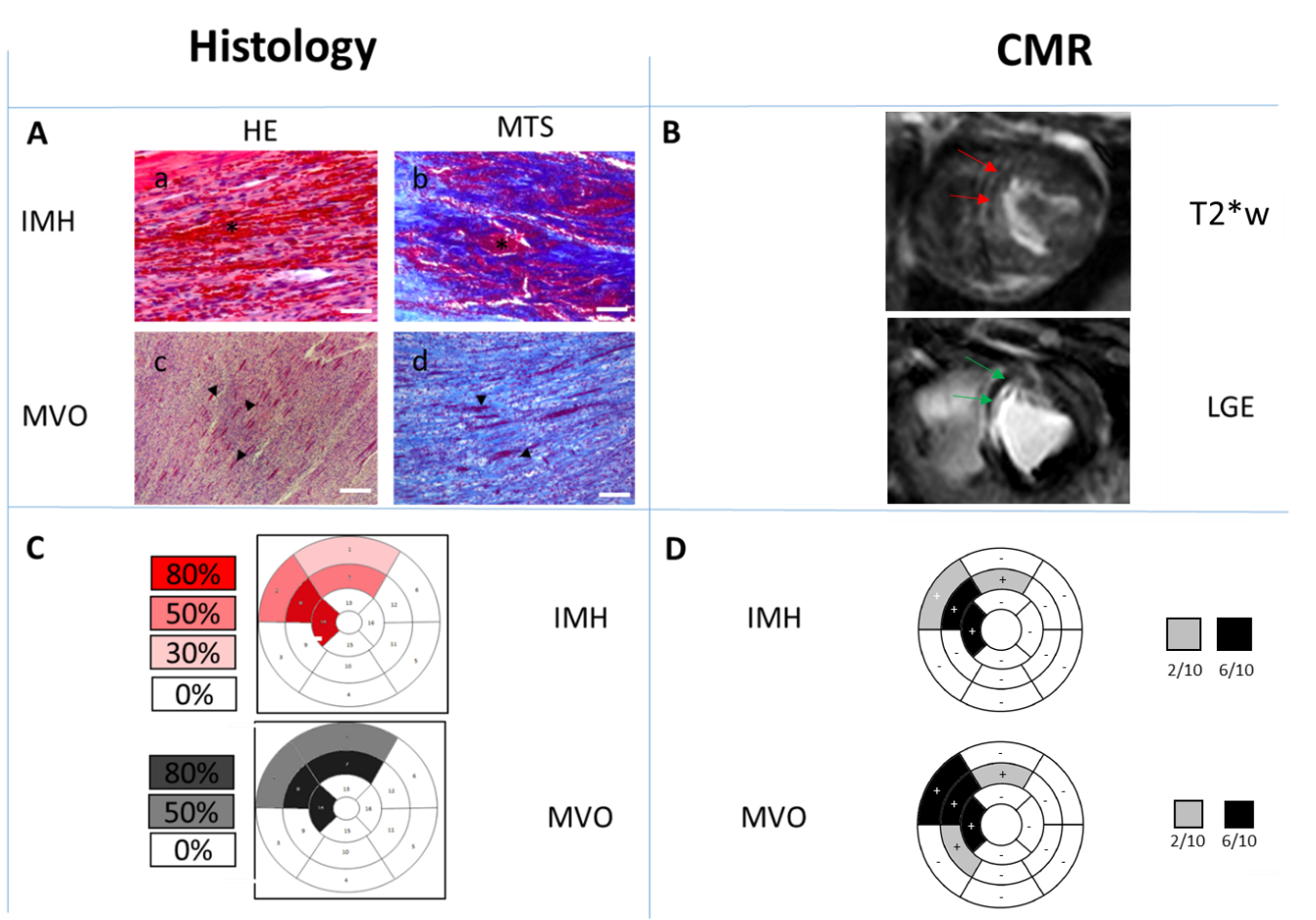


**Figure IV: Histological validation of microvascular obstruction and intramyocardial hemorrhage**

(A) Typical histological slices stained with haematoxylin eosin (HE, a+c) or a Gomori Trichrome Stain (MTS, b+d). Intramyocardial hemorrhage (IMH) (a+b) was defined by vessel and tissue destruction with extravasion of erythrocytes (asterisk) while microvascular obstruction (MVO) (c+d) was defined by plugged microvessels with fibrin, thrombi and erythrocytes (small arrowheads). The regional histological distribution of IMH and MVO throughout the ventricle of all 10 pigs is given in (C) in percentage of the respective segments. (B) IMH seen on T2* weighted (T2*w) cardiovascular magnetic resonance (CMR) images (red arrow) and MVO seen on late gadolinium enhanced (LGE) images (red arrow). The regional distribution of CMR pattern of IMH and MVO throughout the ventricle of all 10 pigs is given in (D) in percentage affection of the respective segments.


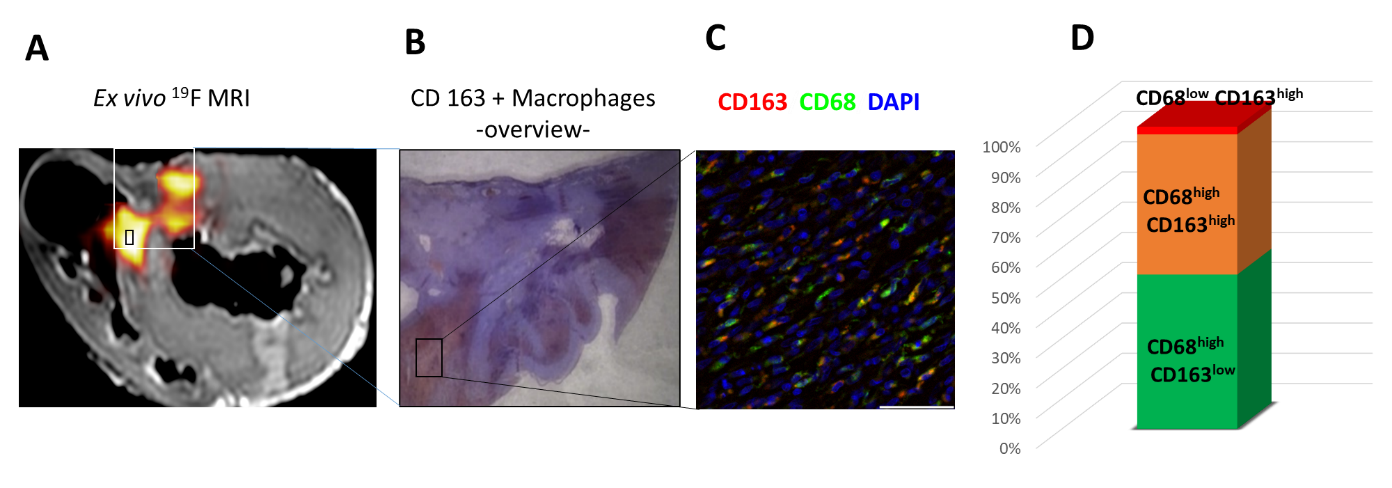


**Figure V: Subdifferentiation of the** **monocyte/macrophage population**

(A) Example of *ex vivo* ^19^F MRI images from an infarcted heart, a region with high ^19^F signal intensity. (B) In direct comparison, a macrophage-rich region was identified in histological slices. (C) Representative picture of fluorescence-stained monocyte/macrophage population in the infarct area with antibodies against CD163 (red, pseudocolored) and CD68 (green, pseudocolored). Nuclei were counterstained with DAPI (4′,6-Diamidin-2-phenylindol). Scale bar 50µm. As given in the analysis (D), the M1 population CD68^high^ CD163^low^ (51±4%) and the M2 population CD68^high^ CD163^high^ (46±5%) were present at nearly equal numbers in all pigs (n=10).


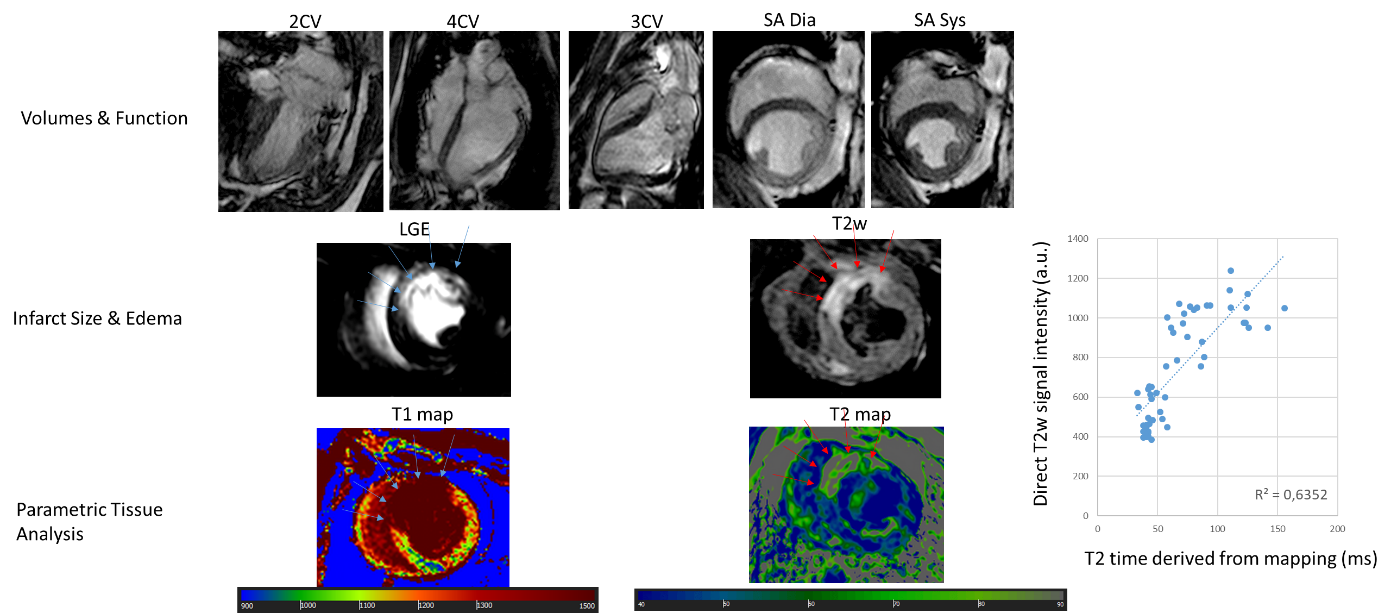


**Figure VI:** **Image quality for ventricular volumes, function, and infarct tissue analysis**

The top row displays typical end-diastolic images acquired in 2, 4 and 3 chamber view (CV) and a midventricular short axis (SA) slice with typical appearance in diastole (Dia) and systole (Sys). Mean row displays typical images after late gadolinium enhancement (LGE) and after T2 weighted (T2w) imaging. Bottom row displays typical parametric maps for T1 and T2 relaxation as acquired with MOLLI and GRaSE Sequences, as given in detail in the Table 1. Regions of myocardial infarction are highlighted by blue (T1-map, LGE) and red (T2-map, T2w) arrows, scaling bars for color-coding of relaxation times are given below the respective map. T2 values were compared with arbitrary intensity units of T2w imaging to account for the quality of T2w image analysis using the indicated threshold method.

**
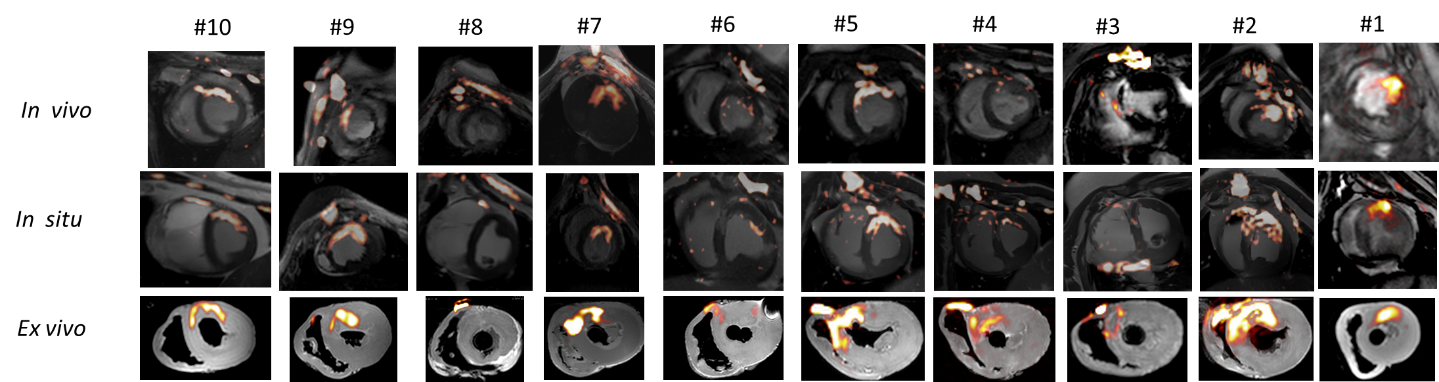
**

**Figure VII:** **Fused ^1^H and ^19^F image examples of all ten pigs**

Shown are midventricular slices of all surviving pigs after cardiovascular magnetic resonance examination under condition *in vivo, in situ, ex vivo*. Each image is a fusion of ^1^H and ^19^F data sets to demonstrate the interindividual distribution and pattern.

$${SNR}_{Z direction}= \frac{0.45\cdot{SNR}_{measured}}{e^{-\frac{Distance in Z direction}{75 mm}}}$$

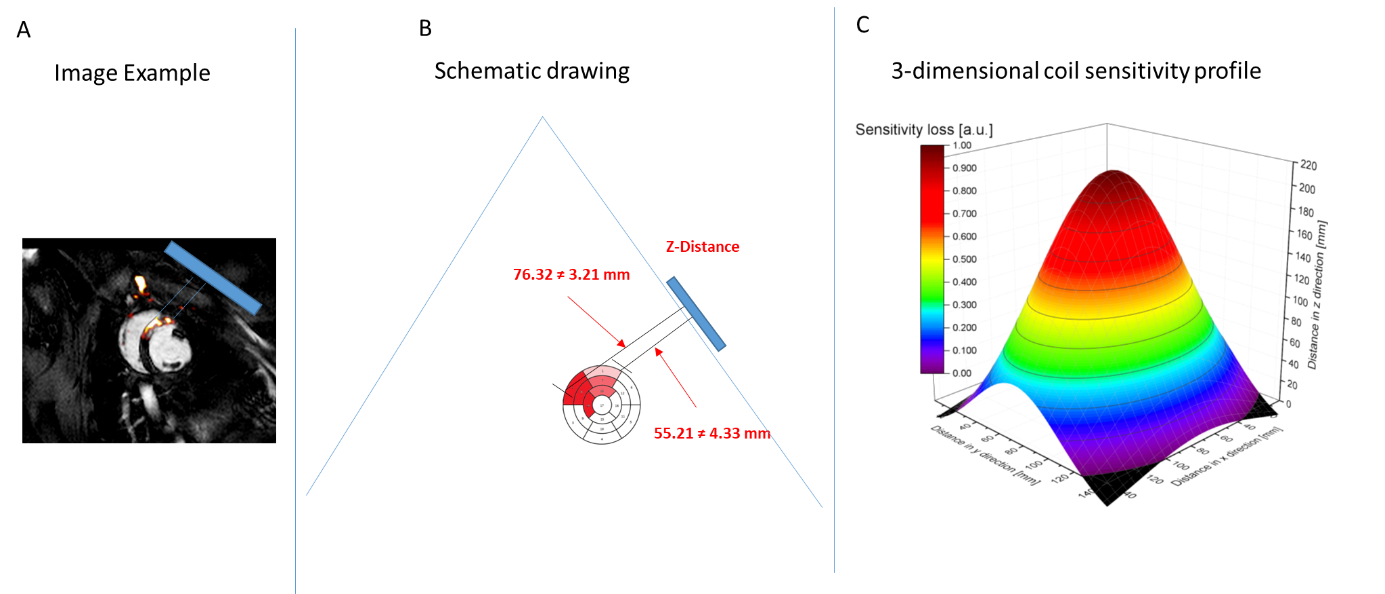


**Figure VIII:** **Signal quantification according to coil sensitivity profile**

(A) The image example illustrates the *in vivo* situation: The coil is placed on the skin of the pig thorax resulting in different depths of myocardial ^19^F signals depending on the pig´s size and thorax shape. (B) On average, the coil distance to the anterior myocardial segments was 55 mm and to the inferoseptal myocardial segments 76 mm. The measured 3D coil sensitivity profile (C) resulted in a comparable signal loss of around 30 % between 5 - 7 cm while deviations in X and Y direction were negligible. True signal intensity, corrected for the coil profile, was calculated as given above.


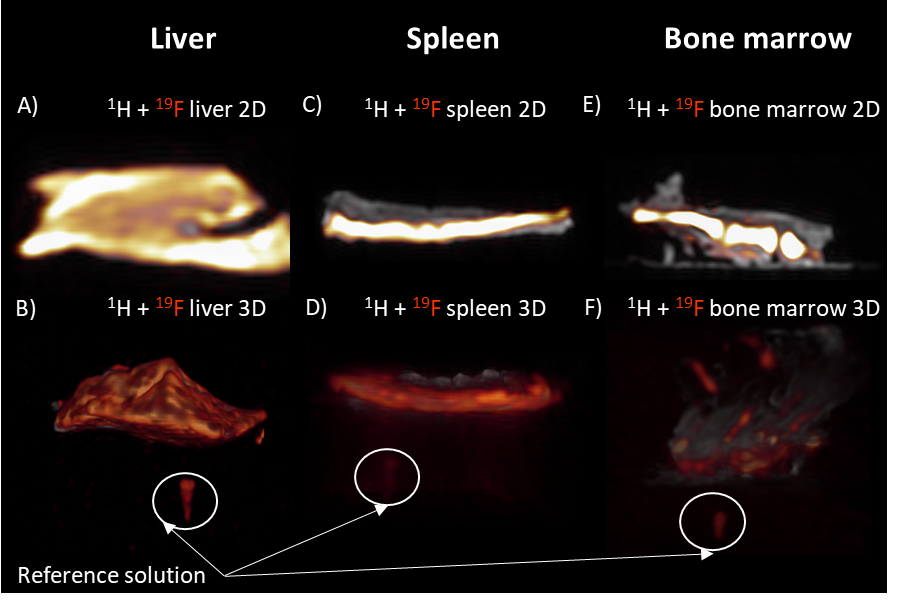


**Figure IX:** **^19^F signal distribution in the reticulo-endothelial system**

Image overlays of ^1^H and ^19^F in 2D of liver (A), spleen (C) and sternum including bone marrow (E) signals. Below the corresponding 3D visualizations of ^1^H and ^19^F signals of liver (B), spleen (D) and bone marrow (F). SNR´s for the three different organs were measured in 3 pigs and showed in liver 10 ± 9, in spleen 22 ± 32 and in bone marrow 4 ± 3 (mean ± SD). A coil-integrated reference solution for precise comparison between signal intensity of different ^19^F images is visible in the 3D visualizations.


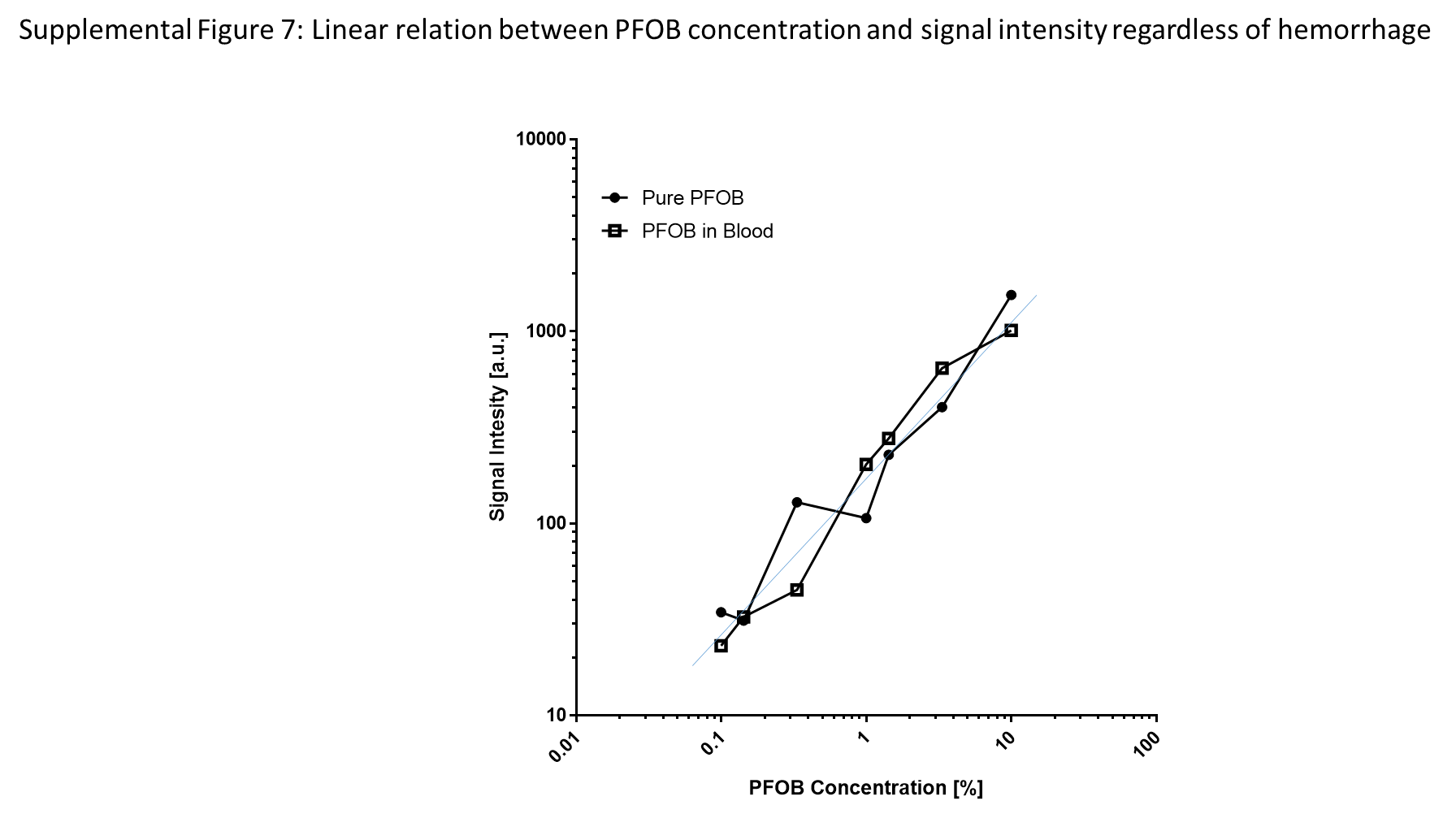


**Figure X:** **Correlation of ^19^F signal intensity and perfluorooctyl bromide- nanoemulsion concentration irrespectively of blood contamination**

The correlation analysis of ^19^F signal intensity and PFOB concentration with (squares) and without blood contamination (circles) yielded R² = 0.9885 for pure and R² = 0.913 for blood contamination, with p<0.01 each. As can be seen, blood had no significant effect on the linear relation of intensity and PBOB concentration.

**
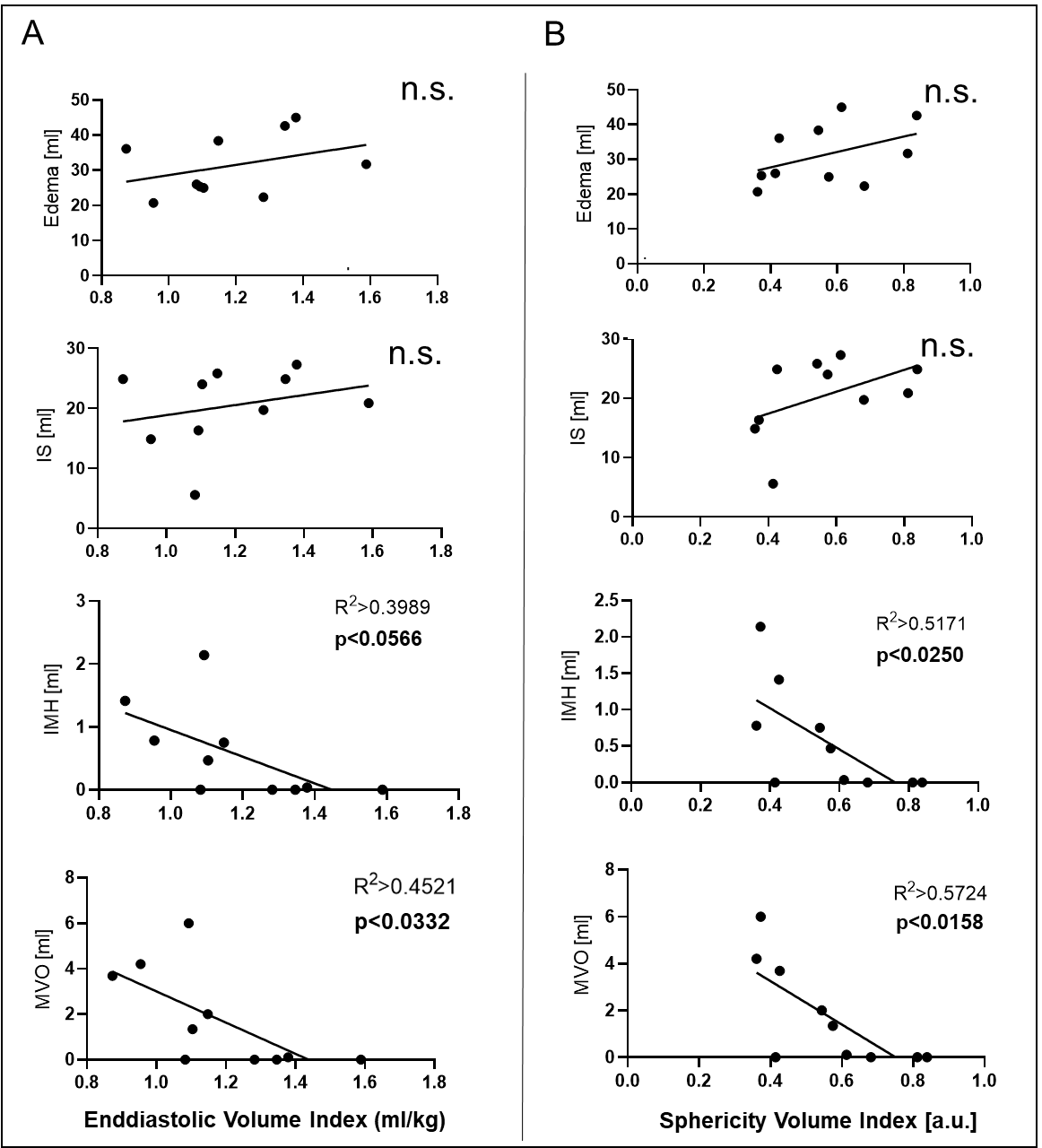
**

**Figure XI:** **Association of infarct tissue characteristics with indexes of initial ventricular remodeling**

Correlation of infarct tissue characteristics (edema, IS=infarct size, MVO= microvascular obstruction, IMH= intramyocardial haemorrhage) as given in millilitre (ml) with indexes of early LV remodelling: (A) enddiastolic volume index and (B) Spericity volume index. The analyses were performed with Pearson`s (Edema, IS) or Spearman`s (MVO, IMH) correlation.

References

1. Ambale-Venkatesh B, Yoneyama K, Sharma RK, Ohyama Y, Wu CO, Burke GL, Shea S, Gomes AS, Young AA, Bluemke DA, Lima JA (2017) Left ventricular shape predicts different types of cardiovascular events in the general population. Heart 103:499-507 doi:10.1136/heartjnl-2016-310052

2. Garcia-Prieto J, Villena-Gutierrez R, Gomez M, Bernardo E, Pun-Garcia A, Garcia-Lunar I, Crainiciuc G, Fernandez-Jimenez R, Sreeramkumar V, Bourio-Martinez R, Garcia-Ruiz JM, Del Valle AS, Sanz-Rosa D, Pizarro G, Fernandez-Ortiz A, Hidalgo A, Fuster V, Ibanez B (2017) Neutrophil stunning by metoprolol reduces infarct size. Nat Commun 8:14780 doi:10.1038/ncomms14780

3. Jensch PJ, Stiermaier T, Reinstadler SJ, Feistritzer HJ, Desch S, Fuernau G, de Waha-Thiele S, Thiele H, Eitel I (2022) Prognostic relevance of peri-infarct zone measured by cardiovascular magnetic resonance in patients with ST-segment elevation myocardial infarction. Int J Cardiol 347:83-88 doi:10.1016/j.ijcard.2021.11.017

4. Kumar A, Green JD, Sykes JM, Ephrat P, Carson JJ, Mitchell AJ, Wisenberg G, Friedrich MG (2011) Detection and quantification of myocardial reperfusion hemorrhage using T2*-weighted CMR. JACC Cardiovasc Imaging 4:1274-1283 doi:10.1016/j.jcmg.2011.08.016

5. Pawlowsky K, Ernst L, Steitz J, Stopinski T, Kogel B, Henger A, Kluge R, Tolba R (2017) The Aachen Minipig: Phenotype, Genotype, Hematological and Biochemical Characterization, and Comparison to the Gottingen Minipig. Eur Surg Res 58:193-203 doi:10.1159/000471483

6. Reinstadler SJ, Stiermaier T, Reindl M, Feistritzer HJ, Fuernau G, Eitel C, Desch S, Klug G, Thiele H, Metzler B, Eitel I (2019) Intramyocardial haemorrhage and prognosis after ST-elevation myocardial infarction. Eur Heart J Cardiovasc Imaging 20:138-146 doi:10.1093/ehjci/jey101

7. Rothe M, Jahn A, Weiss K, Hwang JH, Szendroedi J, Kelm M, Schrader J, Roden M, Flogel U, Bonner F (2019) In vivo (19)F MR inflammation imaging after myocardial infarction in a large animal model at 3 T. MAGMA 32:5-13 doi:10.1007/s10334-018-0714-8

8. Schulz-Menger J, Bluemke DA, Bremerich J, Flamm SD, Fogel MA, Friedrich MG, Kim RJ, von Knobelsdorff-Brenkenhoff F, Kramer CM, Pennell DJ, Plein S, Nagel E (2020) Standardized image interpretation and post-processing in cardiovascular magnetic resonance - 2020 update : Society for Cardiovascular Magnetic Resonance (SCMR): Board of Trustees Task Force on Standardized Post-Processing. J Cardiovasc Magn Reson 22:19 doi:10.1186/s12968-020-00610-6
